# Comprehensive identification of translation start sites by tetracycline-inhibited ribosome profiling

**DOI:** 10.1093/dnares/dsw008

**Published:** 2016-03-23

**Authors:** Kenji Nakahigashi, Yuki Takai, Michiko Kimura, Nozomi Abe, Toru Nakayashiki, Yuh Shiwa, Hirofumi Yoshikawa, Barry L. Wanner, Yasushi Ishihama, Hirotada Mori

**Affiliations:** 1 Institute for Advanced Biosciences, Keio University, Tsuruoka, Yamagata 997-0017, Japan; 2 Graduate School of Pharmaceutical Sciences, Kyoto University, Sakyo-ku, Kyoto 606-8501, Japan; 3 Graduate School of Biological Sciences, Nara Institute of Science and Technology, Ikoma, Nara 630-0101, Japan; 4 Genome Research Center, NODAI Research Institute, Tokyo University of Agriculture, Tokyo 156-8502, Japan; 5 Department of Bioscience, Tokyo University of Agriculture, Tokyo 156-8502, Japan; 6 Department of Microbiology and Immunobiology, Harvard Medical School, Boston, MA 02115, USA

**Keywords:** ribosome profiling, translation initiation, TetRP, genome annotation, N-terminal

## Abstract

Tetracycline-inhibited ribosome profiling (TetRP) provides a powerful new experimental tool for comprehensive genome-wide identification of translation initiation sites in bacteria. We validated TetRP by confirming the translation start sites of protein-coding genes in accordance with the 2006 version of *Escherichia coli* K-12 annotation record (GenBank U00096.2) and found ∼150 new start sites within 60 nucleotides of the annotated site. This analysis revealed 72 per cent of the genes whose initiation site annotations were changed from the 2006 GenBank record to the newer 2014 annotation record (GenBank U00096.3), indicating a high sensitivity. Also, results from reporter fusion and proteomics of N-terminally enriched peptides showed high specificity of the TetRP results. In addition, we discovered over 300 translation start sites within non-coding, intergenic regions of the genome, using a threshold that retains ∼2,000 known coding genes. While some appear to correspond to pseudogenes, others may encode small peptides or have previously unforeseen roles. In summary, we showed that ribosome profiling upon translation inhibition by tetracycline offers a simple, reliable and comprehensive experimental tool for precise annotation of translation start sites of expressed genes in bacteria.

## 1. Introduction

Recent advances in DNA sequencing has permitted rapid determination of the complete genomes of thousands of bacteria.^1^ Computational analyses of these genomes by ORF scan and homology to known genes have greatly facilitated the annotation of protein-coding genes.^2^ However, precise identification of the N-termini of ORFs has proven to be difficult, as documented from the continuous re-annotation of *Escherichia coli* K-12. In early 2006, Riley et al.^3^ reported innumerable updates to gene annotations with 100 s of start site changes, which were supported by a variety of experimental, computational, and database resources. Yet, the 2014 *E. coli* K-12 MG1655 annotation record (NCBI Reference Sequence: NC_000913.3) included 223 start site changes from the 2006 GenBank record, including 133 new start sites for functional genes and 90 start sites for pseudogenes, which were compiled from various newer experimental results and by comparisons with related genomes.^4^

Ribosome profiling (RP) is an application of high-throughput sequencing, in which mRNA protected from RNase digestion by bound ribosomes is used as the source of the sequence library and consequent identification of translated regions of expressed genes.^5–7^ Indeed, RP has provided a wealth of data on translation efficiency,^5,8^ while also changing our understanding of translational control and revealing unexpected translated regions such as upstream micro ORFs and N-terminal identifications.^9,10^ However, RP has not been sufficient for precise identification of translation start sites, at least not in bacteria.

With the goal towards describing translation efficiency in *E. coli*, we conducted RP in the presence of the translation inhibitors chloramphenicol (Cm) and tetracycline (Tet),^11^ which were chosen because they inhibit translation differently.^12,13^ Cm blocks translation elongation by targeting the peptidyl transferase centre on the large ribosomal subunit while Tet inhibits translation by preventing the stable binding of tRNA to the ribosome by directly overlapping with the anticodon stem-loop of tRNA at A-site. While Cm and Tet produced similar RP patterns within central regions of protein-encoding genes, they produced dramatically different patterns near known translation start sites. Importantly, Cm produced broad high-density peaks from the initiation codon to ∼50 nucleotides downstream of the coding region, while Tet produced an RP pattern in which nearly one-half of the signal was sharply concentrated at the location where the initiation codon was at P-site of the ribosome, corresponding to the location of the translating initiation complex.^11^ These results suggested the hypothesis that Tet-inhibited RP (TetRP) may be a powerful new tool for comprehensive and precise identification of translation start sites in bacteria.

Here we validated the usefulness of TetRP for experimental determination of translation start sites and identification of previously unknown translated regions. Results from analysing our TetRP data^11^ confirmed the utility of TetRP for defining functional translation initiation codons.

## 2. Materials and methods

### 2.1. Strains and culture conditions

*Escherichia coli* K-12 BW25113 and its derivatives^14,15^ were used throughout. LB medium^16^ and LB agar were used as rich media. Glucose MOPS medium^17^ prepared as described^18^ was used as minimal medium.

### 2.2. Ribosome profile data used and data processing

The ribosome profile dataset used here has been published^11^ and is available at DDBJ as BioProject ID:PRJDB2960. In brief, samples were taken from glucose-limited continuous cultures of *E. coli* K-12 BW25113 and its *smpB* deletion mutant^14^ in glucose MOPS medium after 30 s treatment with Cm (100 μg/ml) or Tet (40 μg/ml). RP following treatment with clindamycin (10 µg/ml; clindamycin hydrochloride monohydrate, Tokyo Chemical Industry, Japan) and pactamycin (5 µg/ml, Sigma) were similarly performed using an *E. coli* K-12 BW25113 *tolC* mutant,^14^ which is more sensitive to these antibiotics.^19,20^

Because the sequence libraries were constructed by adding polyA to the 3′-ends of the short RNA fragments produced with RNase I, the polyA sequences were computationally eliminated using fastx_clipper in the FASTX-Toolkit (http://hannonlab.cshl.edu/fastx_toolkit/index.html, accessed 27 February 2016) and mapped to the *E. coli* K-12 genome (GenBank: U00096.2) using bowtie.^21^ Number of reads mapped to coding region was about one to a few million (Supplementary Table S1). Mapped length of reads distributes mainly from 25 to 50 nucleotides, due to size selection of digested mRNA fragments by polyacrylamide gel electrophoresis.^11^ The length distribution of TetRP reads mapped to CDS, indicating protected length by ribosome, is shown in Supplementary Fig. S1. To compare the read depth at each genomic position, the mapping information of each read was used to summarize the read depth at each position on both strands. For precise mapping, positions corresponding to 3′ ends of reads were used for calculation. The read depth was normalized between samples by defining the average depth over the entire coding region as one; the resultant depth was used as the signal strength at each position. Because 3′ adenines were removed during data processing, reads ending before and after a 3′ adenine were counted as the same position, depth at this position was used for both positions. While this process reduced resolution, we found this step was a reasonable solution for purposes of data analysis.^11^ Though the protected length by ribosome ranges mainly from 25 to 50 nucleotides in the datasets, our previous analyses showed that it extends 12- to 13-nt in the 3′-direction from the first base of A-site codon but various lengths in the 5′-direction;^11^ thus, an average read depth over 15- to 16-nt 3′ was used as the signal to identify codons starting from a specific genome position.

### 2.3. Screening new translation start sites of known coding genes

In-frame NTG codons <60-nt 5′ from the initiating codon of coding genes^3^ were listed, and signal ratio of the codon to the corresponding new start codon was calculated after addition of 0.5 to both values to avoid division by zero. Candidates for alternate start codons were then selected as described in Results.

### 2.4. Construction of β-galactosidase gene fusions to confirm new translation start sites

The modified *lacZ* DNA gene behind the *lacUV5 promoter* without an operator from pKK232-Z (GGA)^22,23^ was replaced with the *lacZ* alpha region from the low copy number plasmid pMW228 (Nippon Gene Co. Ltd., Tokyo), to construct the low copy number plasmid pMW-base-lacZ, which expresses *lacZ* constitutively. For this, PCR fragments were generated using pMW228 and pKK232-Z(GGA) as templates and primers XbaI-pMW218-1874R ccttctagACAGCTTTGAATGCACCAAA with XhoI-pMW218-2570F ctcctcgagTTTCTCATAGCTCACGCTGT, and XbaI-TP17-F ccttctagAATTCAGCCCGCCTAATGAG with XhoI-rrnBterm ctcctcgagTGCTTTCCTGATGCAAAAAC, respectively, digested with XbaI and XhoI and ligated. *lacZ* gene fusions were made by replacing DNA from the BamHI site downstream of the *lacZ* transcription start site to 6th codon of *lacZ* with the test sequence (Supplementary Fig. S2). To confirm translation was initiated from the test sequence, the central base of either or both possible translation start codons in the region were changed from T to C. All segments generated by PCR were confirmed by DNA sequencing of the relevant regions in the respective plasmids.

### 2.5. β-galactosidase assays

*Escherichia coli* K-12 BW25113 harboring each plasmid was grown aerobically in minimal medium containing 0.4% glucose and 25 µg/ml ampicillin to 0.5 OD_660_ as measured with a digital spectrophotometer (miniphoto 518R, Taitec Corp, Japan). Cultures were cooled on ice and assayed for β-galactosidase as described.^24^ Averages and standard deviations from triplicated cultures were determined.

### 2.6. Proteomics of N-terminal peptides

*Escherichia coli* K-12 BW25113 was grown to mid-log phase in LB broth with vigorous shaking at 37°C. Six replicated cultures were analysed as follows. Cells were collected by centrifugation and re-suspended in buffer containing 100 mM Tris–HCl (pH 9.0), 12 mM sodium deoxycholate, and 12 mM sodium lauroyl sarcosinate. The protein crude extract was treated by reductive dimethylation followed by trypsin digestion. Protein N-terminal peptides were enriched by COFRADIC^25^ and desalted with a SDB-XC StageTip (GL Sciences, Tokyo, Japan).

NanoLC-MS/MS analyses were performed on TripleTOF 5600 (AB SCIEX) system, connected to a Thermofisher Scientific UltiMate 3000 pump (Germering, Germany) and a HTC-PAL autosampler (CTC Analytics). Peptides were separated in a self-pulled needle column (150 mm length × 100 µm ID, 6 µm opening) packed with Reprosil-C18 3 μm reversed-phase material (Dr Maisch GmbH, Germany). The mobile phases consisted of (A) 0.5% acetic acid and (B) 0.5% acetic acid and 80% acetonitrile. A three-step linear gradient of 5–10% B in 5 min, 10–40% B in 60 min, 40–100% B in 5 min, and 100% B for 10 min was employed. The mass scan ranges were *m/z* 300–1,500, and top 10 precursor ions were selected in each MS scan for subsequent MS/MS scans.

Peptides and proteins were identified by automated database searching using Mascot v2.3 (Matrix Science, London, UK) with a precursor mass tolerance of 20 ppm, a fragment ion mass tolerance of 0.1 Da, and strict trypsin specificity allowing for two missed cleavages. The protein database searched was produced from the 2006 *E. coli* K-12 MG1665 genome record,^3^ with or without an update from the TetRP results. Peptides were considered identified if the Mascot score was over the 95% confidence limit (*P* < 0.05) for each peptide. False discovery rate was set to be 1% at peptide level.

### 2.7. Screening for new translation start sites within intergenic regions

ATG codons outside coding regions, excluding ones <30-nt from the 5′-end of coding regions (betMet), were selected from the 2006 *E. coli* K-12 annotation^3^ and used to identify possible new start sites as described in Results.

### 2.8. Construction of genomic Venus fusions to intergenic regions

Venus fusions to predicted intergenic coding regions were made by replacing the termination codon of the targeted ORF with a DNA fragment containing Venus (without a start codon) and chloramphenicol resistance (*cat*) gene by λ Red-mediated recombination.^15^ The Venus-*cat* template plasmid was made by inserting the Venus coding region^26^ into pKD3.^15^ DNA sequences of the template and PCR primers are in Supplementary Fig. S3.

### 2.9. Flow cytometry

Genomic Venus fusion strains were grown in glucose minimum medium to 0.3 OD_600_ or LB broth overnight and subjected to flow cytometry using a FACScan flow cytometer (Becton, Dickinson) as described elsewhere.^27^

## 3. Results

### 3.1. Different patterns of Tet- and Cm-inhibited ribosome profiling

**Figure 1.**
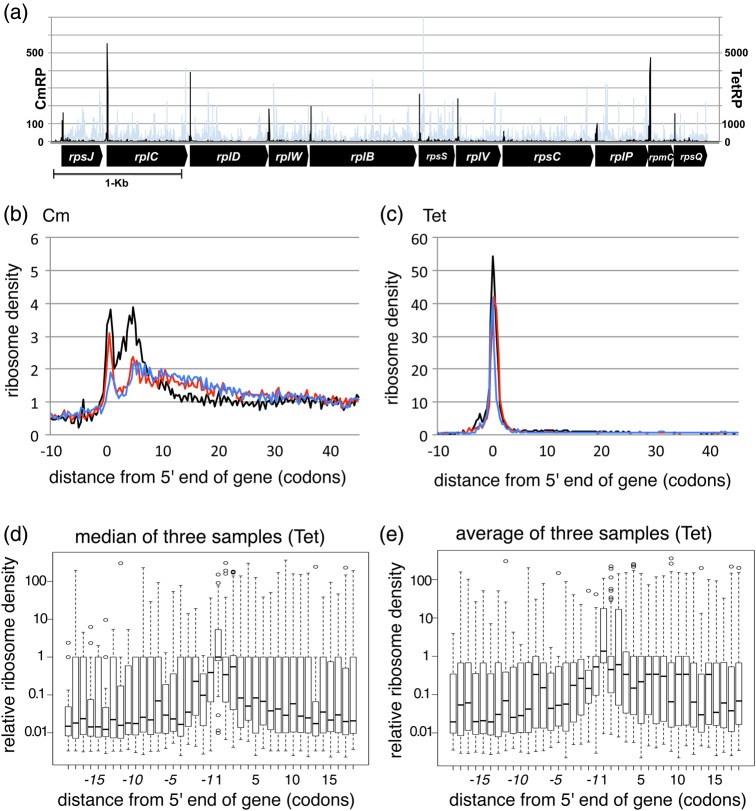
Difference in TetRP and CmRP signal patterns. (a) TetRP and CmRP patterns of a ribosomal protein operon. TetRP (Black) and CmRP (light gray) patterns over an operon coding ribosomal proteins are shown. Location of each gene is shown by an arrow. (b) CmRP and (c) TetRP patterns near 5′ end of coding region of genes. Genes longer than 500-bp were selected, and read depths from −5 to +495 from the first base of initiation codon were normalized to an average value of the region, then the depth of all genes was averaged. Results from three independent cultures are shown in different colours. (d, e) Relative TetRP signal distribution at in-frame ATG codons near initiation codons of known genes is compared with signals at the corresponding initiation codon. (d) Median and (e) average signals at a position from three samples were used for calculation. Median, upper and lower quartiles of all ATG codons at indicated distances are boxed. This figure is available in black and white in print and in colour at *DNA Research* online.

In an earlier study, we found huge differences in RP read patterns between samples from Cm- and Tet-treated cultures. Whereas Cm-inhibited RP (CmRP) patterns show signals distributed to all coding regions, TetRP patterns have sharp peaks at translational initiation sites (Fig. 1a). At around initiating sites, Cm-inhibited RP (CmRP) patterns show broad high-density reads from the initiating codon to ∼50 nucleotides downstream of the coding region, instead TetRP patterns have peaks at positions where the initiating codon is at the ribosome P-site (Fig. 1b and c).^11^ Although the mechanism responsible for these differences is not well understood, the characteristic RP pattern from TetRP samples led us to propose the hypothesis that TetRP may be useful for identification of translational start sites in bacteria, which we tested as described below.

We initially tested our TetRP hypothesis by comparing TetRP reads at initiating codons with RP reads at nearby in-frame AUG codons (alt-AUG) in known coding genes. Figure 1d and e shows the ratio (reads at alt-AUG codons)/(reads at corresponding initiating codons) against the relative position to the initiating codon. Results showed that read depths at initiating codons were >10-fold higher than at most distal positions. Differences were less at closer positions, and depths at +1 positions (the second codon following the initiating codons) were almost the same as at the initiating codon, possibly due to functioning of alternative initiating codons, the limits of resolution of our data, or both.

### 3.2. Re-mapping start sites of known *E. coli* protein-encoding genes

Based on the results above, we attempted to re-annotate the starts of all coding genes, noting that by nature RP data pertain only to genes expressed under experimental growth conditions. We did this by comparing our results with the 2006 annotation record of the *E. coli* K-12 MG1655 genome (GenBank U00096.2), but excluding pseudogenes and IS genes.^3^ By comparing read depth at initiating codons with nearby (<60-nt) in-frame NUG codons, we selected 177 (1.2% of 17,319) possible alternative initiating (alt-init) codons, which showed higher read depth in two of three samples (median ≥1) and ≥10-fold higher read depth on average. These 177 sites correspond to 165 genes, including 10 genes with 2 new start site candidates and 1 gene with 3 start site candidates. Also, 11 alt-init sites lying upstream of the 2006 annotated site have in-frame stop codons preceding the annotated initiating codon. The remaining 154 genes are listed as alt-init codon(s) in Supplementary Table S2.

**Figure 2.**
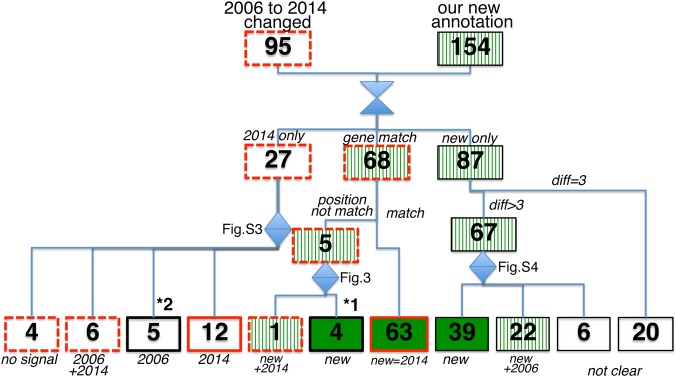
Confirmation flowchart of possible new initiation sites of known genes. Boxes with dark gray (red) lines indicate genes changed annotation in 2014 annotation record; dashed lines indicate unconfirmed or inconclusive changes. Shadowed boxes (green) indicate genes changed by our analysis; stripes indicate unconfirmed or inconclusive changes. Boxes denoted *1 and *2 are 2014 annotation changes that did not match with our analysis. See text for further explanation. This figure is available in black and white in print and in colour at *DNA Research* online.

**Figure 3.**
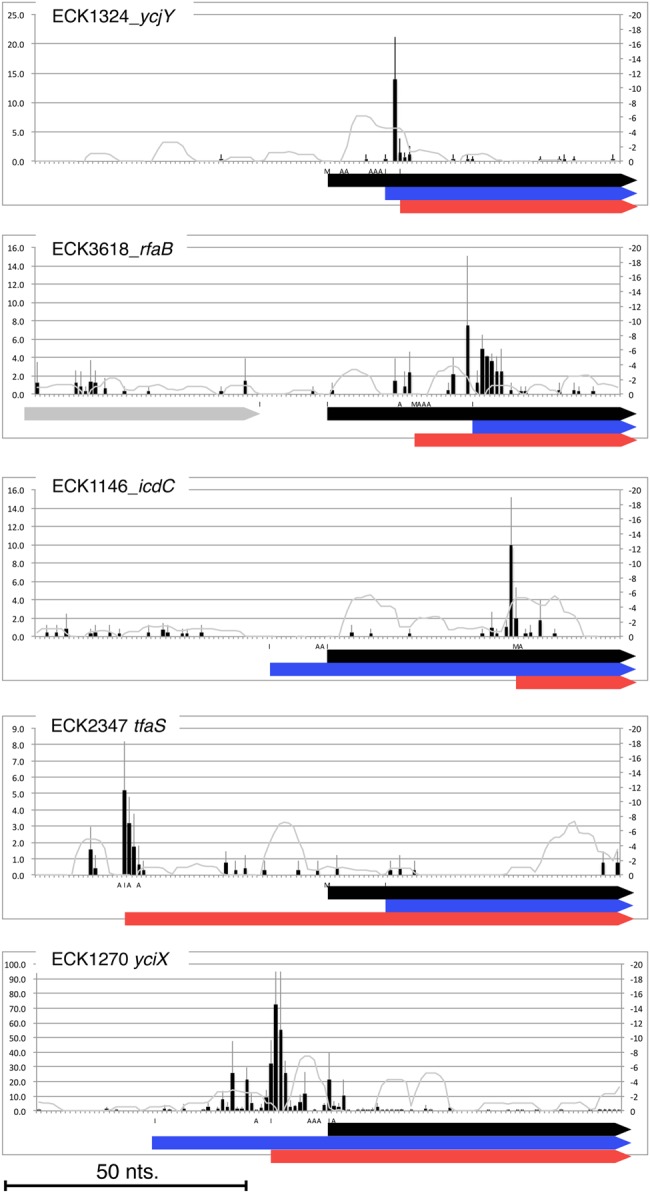
TetRP signals near initiation region of triply annotated genes. An average + SD value of TetRP signal at each genome position is shown by a black bar and a line. (The TetRP signal is shown at 15-nt 5’ from the 3’-end of the read, corresponding to the first base of P-site codon.) The calculated hybridization stability with anti-ribosome binding site of 16S rRNA (ΔG) is shown by gray line. Coding region starting from the 2006 (top, black), 2014 (middle, blue), and TetRP (bottom, red) annotations are indicated as thick arrows and an upstream gene is sown by a thick gray arrow. A, I, or M in X-axis indicates, A: a position 15-nt. 3’, where sequence reads giving the signal at the position, is adenine, I: end or start position of a gene, or M: both of them. This figure is available in black and white in print and in colour at *DNA Research* online.

We next compared the TetRP data with the 2014 *E. coli* K-12 MG1655 annotation record (GenBank U00096.3), which was released while this study was in progress. The 2014 GenBank record has 95 genes with re-annotated start sites within our search range, including 63 that match our TetRP results (Fig. 2 and Supplementary Table S3); 5 other genes have new start sites by our analyses, which differ from the re-annotated start sites. Figure 3 shows the read depth near the start sites of these 5 genes. Inspection of the signal at three possible initiating sites show that the major signals for *ycjY*, *icdC*, *yciX* (*ymiA*), and *tfaS* are at the start site that we mapped and little signal lies at start sites in the 2006 and 2014 *E. coli* K-12 MG1655 annotation records. For *rfaB*, the sites from both the TetRP results and the 2014 annotation show signals. An additional strong signal exists near the 2014 *rfaB* annotated site, indicating that its major initiation site is at this site.

We similarly investigated 27 genes whose start sites were changed from the 2006 to 2014 annotation records and were not revealed by our initial analyses (Supplementary Fig. S4 and Table S3). Upon manual inspection, we found that 12 have reads only at the 2014 annotation start sites. We failed to find these initially due to variability among the triplicate samples. Five have signals only at the 2006 annotation start sites, supporting the validity of our analyses for these genes. Six have signals at both the 2006 and 2014 annotation start sites, suggesting alternative start sites function for these genes. Four have no signal at either start site.

Of the 9 cases where the 2006 to 2014 annotation changes did not match with our analysis (marked *1 and *2 in Fig. 2), two revisions of the 2014 record were made based on proteomics data. In *yciX* (*ymiA*), a peptide not expected from 2006 annotation was found,^28^ but which can be derived from proteins initiated from the 2014 annotation and TetRP site. Accordingly, we were unable to determine whether the 2014 or TetRP site is correct from the peptide data. For *trpC*, an N-terminal peptide corresponding only to the 2014 annotation was found,^28^ contrary to our finding that little TetRP signal was at this site. In seven other cases, the 2006 to 2014 revision appears to have no experimental support (Supplementary Table S3). Thus, excluding these 8 of 9 cases, our TetRP analyses agree with 72% [63/(95 − 8)] of the start sites re-annotated from 2006 to 2014.

We also closely inspected reads near the initiation sites of 87 genes for which we found new start sites and which were unchanged in the 2006–14 annotation records (Fig. 2). We removed 20 cases with one codon difference from these analyses, because the differences in the read depth between the initiation codon and an adjacent ATG codon were small (Fig. 1 d and e). We analysed the remaining 42 ATG, 10 GTG, 9 TTG, and 6 CTG new start sites (Supplementary Fig. S5). Of these 67 cases, 39 (27 ATG, 4 GTG, and 8 TTG) have clear signals mainly at the new start sites; 22 (14 ATG, 5 GTG, and 3 CTG) have signals at both the original and the newly annotated sites (Supplementary Table S2); and 6 cases were inconclusive due to weak signal or broad signal distribution near the initiation site(s). For two genes (*ymdC* and *surE*), we were unable to ascertain whether the signal near the start sites was real due to overlap of the initiation sites by coding regions of strongly expressed upstream genes. Accordingly, 39–61 (39 + 22) of these 67 genes appear to initiate from the newly identified start sites. Our data not only show high validity but also reveal many genes with dual translation initiation sites.

### 3.3. Use of β-galactosidase gene fusions to confirm new translation start sites

**Figure 4.**
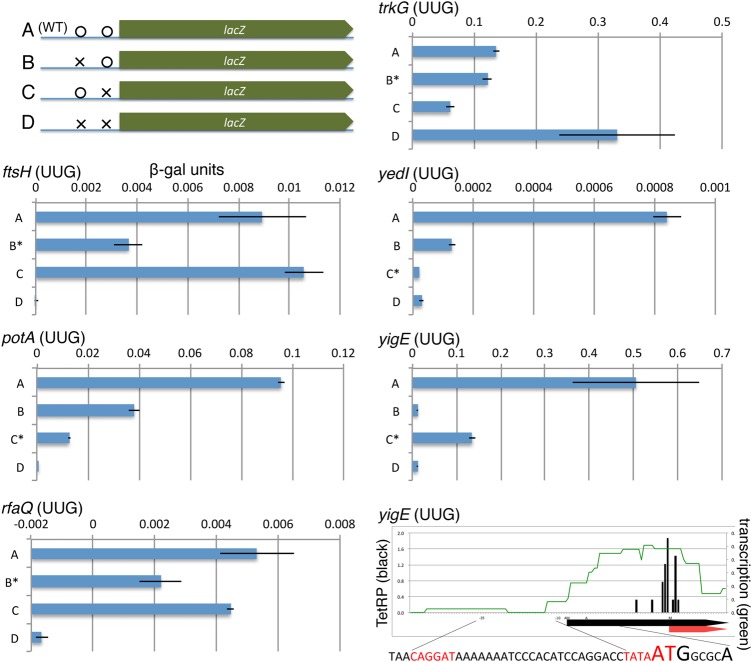
β-Galactoside activity from *lacZ* reporter gene fusions. Results from fusions to initiation regions (A) with both possible translation initiation codons (wild type), (B) with upstream initiation codon mutated, (C) with downstream initiation codon mutated, and (D) with both initiation codons mutated. The construct with the new annotated initiation codon mutated is marked with an asterisk. Upper left panel shows the structures of the gene fusions with an *X* marking mutated sites. Lower right panel shows TetRP (black), transcription (gray), and DNA sequence near the *yigE* initiation codon. -35 and -10 regions of the possible promoter are highlighted (red). The 2006 ATG initiation codon and transcription start site are enlarged. This figure is available in black and white in print and in colour at *DNA Research* online.

It is notable that we found 9 new UUG start sites, as only 81 UUG start codons had been previously annotated.^3^ We therefore confirmed several new minor initiation codons (6 UUG and 1 GUG) by comparing their ability to initiate translation with the originally annotated AUG codon by using *lacZ* reporter gene fusions. To do this, we made a series of *lacZ* fusions to DNA fragments containing both the original and newly identified initiating codons plus 15-nt 5′- and 3′-flanking sequences to *lacZ* without a start codon, in which expression was driven by the *lacUV5* promoter on a low copy number plasmid (Supplementary Fig. S2). We then destroyed either or both initiating codons by mutating its second position T (U) to C. The amounts of β-galactosidase made by the resultant constructs are in Fig. 4. Results show that in four of seven cases (*ftsH*, *potA*, *rfaQ*, and *yedI*) elimination of the new start site (marked by an asterisk in Fig. 4) has a greater effect on reducing β-galactosidase levels than alteration of the original start codon, indicating more efficient translation from the new start codon. In the case of *yigE*, removal of original (upper) initiating codon has a larger effect. However, examination of *yigE* transcription in the culture used for RP revealed that transcription initiates between the original and newly annotated start sites; thus, the start site showing higher translation for the *lacZ* fusion is not used in the genome context (Fig. 4 lower right panel). We were unable to draw a definitive conclusion for two genes because (i) the β-galactosidase levels were too low in all cases for *ybjO* fusions (data not shown) or (ii) the *trkG* fusion unexpectedly showed the highest level in the construct in which both initiation codons altered (Fig. 4). Thus, five of the seven cases, including 4 with UUGs, indicated higher translation for the newly annotated codon than the original AUG codon, while one case (*yigE*) was true only in its genomic context. Our data show that these non-ATG codons function efficiently. It is notable that our newly found UUG start site for *ftsH* had been previously reported,^29,30^ but it had not been included in the 2006 nor 2014 GenBank annotation records.

### 3.4. Confirmation of the new translational initiation start sites with proteomics

We also used proteomics to confirm the newly annotated start sites by selectively enriching N-terminal peptides after tryptic digestion and LC-MS identification of peptides. In this analysis, we found N-terminal peptides corresponding to 910 proteins of the 4,300 total known coding genes, including N-terminal peptides for 19 of the 154 genes with new start sites, counting peptides starting with the initiating methionine or the second amino acid (Supplementary Table S4). We also found N-terminal peptides for 8 of these 154 genes corresponding to the 2006 annotation. Importantly, we found two N-terminal peptides for 5 genes, including one corresponding to our new start site and the other corresponding to the 2006 annotation start site. Peptides corresponding to the new site were found more frequently in four cases, indicating dual translation initiation sites and more efficient translation at the new site. Our discovery rate of 12 per cent (19 N-terminal peptides for 154 genes) is significantly lower (*P* < 0.5) when compared with 21 per cent (910 N-terminal peptides for 4,300 genes) for total proteins. However, considering 60% specificity will result in similar discovery rates indicated good accuracy of our analysis.

### 3.5. Identification of intergenic translation start sites

Having shown TetRP to be an accurate and reliable tool for identification of translation start sites of known genes, we sought to identify translated regions within intergenic (non-coding) regions of the *E. coli* K-12 MG1655 genome in the 2006 GenBank record. We examined reads at 14,942 ATGs between intergenic regions, denoted betMet, excluding ATGs within 30-nt of known start sites, as potential alternative translation initiation sites. We selected intergenic start sites by TetRP signal strength at a threshold of average − standard deviation in log (TetRP signals) of known start sites (initMets), but not using those with no signal. This led to keeping 2,588 of 4,310 initMets but only 409 of the 14,942 betMets (Supplementary Fig. S6a). We then used the TetRP/CmRP ratio at a threshold, keeping 80% (2,071) of the remaining initMets, and selected 360 betMets as candidates for intergenic translation start sites (Supplementary Fig. S6b and Table S5). These 360 intergenic start sites include 27 that were in the 2014 GenBank annotation record (Supplementary Table S5), including 18 newly annotated protein-coding genes, 5 pseudogenes, and 4 new start sites of known genes. We also identified 5 others as new start sites of known genes. Thus, 328 may function as start sites of novel intergenic translation units. As expected from their intergenic locations, most predicted coding regions are short: only 16 are predicted to encode >50 amino acid residues and 80% encode fewer than 24 residues (Supplementary Fig. S6c).

### 3.6. Use of genomic Venus fusions to confirm translation initiation of newly discovered small ORFs

**Figure 5.**
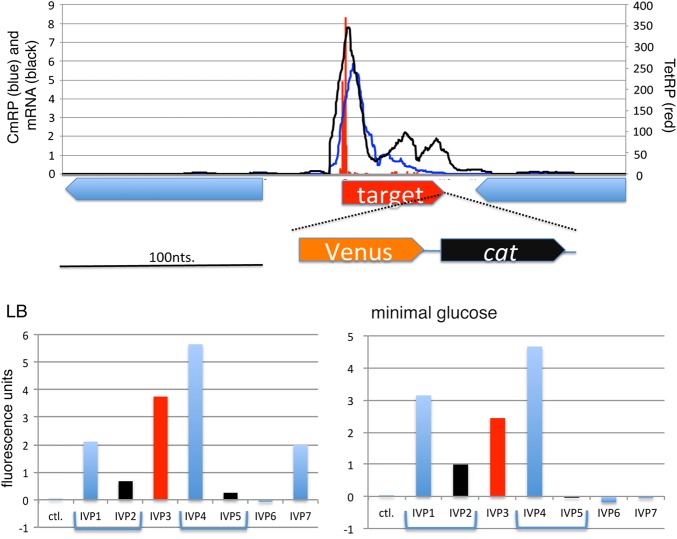
Expression of small (s)ORF-Venus fusion in genomic context. Upper panel: Expression pattern of the sORF in wild type and structure of the fusion gene. TetRP (red, mapped location of 3′-end of sequence reads), CmRP, and transcription (blue and black, respectively; all sequence reads mapped) signals are shown with the location of sRNA and flanking genes. Venus and *cat* DNA cassette was inserted at the 3′ end of sORF as described in Materials and Methods. Lower panels: Fluorescence from the Venus fusion protein in each strain is shown following growth on LB or glucose minimal medium, as indicated. Fluorescence from wild type lacking Venus was used as background. Signal from positive control (IVP3, *yciY*) is in red, and negative controls (IVP2 and 5) are in black. This figure is available in black and white in print and in colour at *DNA Research* online.

To test newly identified intergenic start sites for function in their native genomic context, we constructed a series of Venus (bright yellow fluorescent protein: Ref. 26) gene (translation) fusions to the 3′-end of selected ORFs by recombining a Venus-*cat* cassette into the genome as shown in Fig. 5 (upper panel). We chose five small ORFs including *yciY* (from the 2014 annotation^4,31^) for analysis, which we named IVP1, 3 (*yciY*), 4, 6, and 7 (Supplementary Table S5 and Fig. S7). We also chose two intergenic ORFs with ATGs with lower TetRP signal (IVP2 and 5) than selected, but located 7- and 10-nt from IVP1 and IVP4, respectively, to check the resolution of our analysis. Our flow cytometry data for these strains and a control wild type without Venus are in Fig. 5. On the one hand, in LB medium three of four and in minimal medium two of four fusions to these ORFs produced expression levels similar to the IVP3 (*yciY*) fusion, indicating that these start sites are functional. On the other hand, the IVP2 and IVP5 fusions showed lower fluorescence in both medium than IVP1 and IVP4, respectively, indicating that TetRP reads can discriminate sites separated by 10 or fewer nt.

## 4. Discussion

We showed that TetRP is a reliable experimental tool for comprehensive and precise identification of translation start sites for expressed genes in *E. coli*. It is especially notable that TetRP revealed >70% of start site changes in the latest (2014) GenBank annotation record, which included many sites manually compiled from a number of individual experimental studies in addition to ones identified by homology to closely related genomes,^4^ while also producing a low false discovery rate. The high sensitivity and specificity of finding working initiation sites based on existing annotation data shows TetRP to be reliable for validation of correct translation start sites, following standard gene annotation steps of ORF scan and homology searching.

RP following treatment with translation inhibitors has been previously used to identify translation initiation sites in eukaryotes,^10,32^ but not in bacteria. We tested several antibiotics in addition to Cm and Tet and found two others (clindamycin and pactamycin) that showed similar initiation site patterns as Tet (Supplementary Fig. S8). Since pactamycin is believed to inhibit the first translocation from initiation complex,^12^ emergence of initiation site-specific RP pattern for pactamycin is expected. However, clindamycin and Tet inhibit translation elongation by binding to 50S subunit to inhibit peptidyl transferase reaction and to 30S subunit to block aminoacyl-tRNA entry, respectively.^12,33,34^ More information on the mode of action is required to understand the basis of the observed initiation site-specific RP patterns. Irrespective of full understanding, three antibiotics are applicable for identification of translation start sites, though Tet is generally more suitable considering its broad specificity and availability.^12^ As described herein, the resolution of TetRP in this report was somewhat limited by the addition of polyA to short RNAs prior to reverse transcription during the construction of sequencing libraries, which led to an ambiguity of the 3′-end positions and required sequencing in the 5′ to 3′ direction with respect to the RNA to find 3′-ends.^11^ While we used this method to avoid quantitative bias in RNA sequencing,^5^ the use of adapter ligation to 3′-ends of short RNA for library construction and sequencing in the 3′ to 5′ direction of the RNA would be useful to enhance resolution.

With regards to genome annotation of *E. coli* K-12, results in this work are important not only because we found many new N-termini of known proteins in the extensively re-annotated 2014 GenBank record annotation,^4,35^ but also because we uncovered multiple translation initiation sites for many genes. We identified 28 genes with two initiation sites by TetRP, including 5 proteins with two start sites by N′-proteomics. Although, only a few cases had been found in *E. coli*,^36,37^ proteins with multiple start sites may be more prevalent than previously had been thought. Our results from testing alternate initiation codons by constructing β-galactosidase gene fusions also showed that in most cases constructs lacking either initiation codon retained some activity while constructs lacking both codons showed lowest activity (Fig. 3), which also suggests functioning of multiple initiation codons. These genes may change the major translation initiation site in response to cellular environments or signals, and TetRP can be a useful means to globally investigate mechanisms of how cells select translation initiation site.

We also found evidence for >300 new translation units without using homology or ORF scan information. Some such ORFs are likely to have function like other small proteins recently found in the cell membrane^38,39^ or are pseudogene fragments like ones that have already been included in the 2014 *E. coli* K-12 GenBank record (NCBI Reference Sequence: NC_000913.3). However, it is difficult to infer how many of the 328 new intergenic translation units have biological function. Though TetRP signal strength of these possible initiation sites are generally similar with the known initiation sites, many weak sites may result from low level infidelity of ribosomes binding to RNA and the high sensitivity of TetRP to detect such binding sites. For example, we recently uncovered thousands of previously undocumented transcription start sites in *E. coli* K-12 by strand-specific RNA-seq,^40^ many of which are antisense to coding regions. Whether these have biological roles or correspond to genomic ‘dark matter’ is unknown.^41^ In support of this notion, we found that the length distribution of ORFs from the intergenic start sites was similar to the length distribution of all ORFs within intergenic regions or ORFs randomly produced by the genetic code (Supplementary Fig. S6c). Accordingly, peptides produced by these new initiation sites appear not to have been selected by their function. It is also notable that the portion of very short ORFs (<9 residues) is higher in the selected ORFs than in the random or total ORF sets. Especially, the portion of single-codon ORFs, i.e. ones with a start codon followed immediately by a stop codon was higher in the selected ORFs. Translation of the detected small intergenic ORFs may have biological function even if the translation product has no function. Ribosome binding or translation of short ORFs may have key gene regulatory roles in both prokaryotes and eukaryotes.^42–45^ Yet, many of the intergenic translation initiation sites were not obviously related to nearby coding genes, though ribosome binding to such RNAs may affect its function by changing its structure or stability.^46^

## Supplementary data

Supplementary data are available at www.dnaresearch.oxfordjournals.org.

## Funding

This work was supported by Japan Society for the Promotion of Science Grant-in-Aid for Scientific Research (B) (20310117 and 24310148 to K.N.), (A) (25250028 to H.M.), and Grant-in-Aid for Scientific Research on Innovative Areas (26116717 to H.M and K.N.); the National Science Foundation (106394 to B.L.W.); and a co-operative research grant of the Genome Research for Bioresource, NODAI Genome Research Center, Tokyo University of Agriculture. Funding to pay the Open Access publication charges for this article was provided by Japan Society for the Promotion of Science, Grant-in-Aid for Scientific Research (A) (25250028).

## Supplementary Material

Supplementary Data
