# Supplementary material for: Comprehensive identification of translation start sites by tetracycline-inhibited ribosome profiling
Source: DNA Res. 2016 Mar 23;23(3):193–201. doi: 10.1093/dnares/dsw008 (PMC4909307; doi:10.1093/dnares/dsw008)
Supplement: Supplementary Data [file supp_23_3_193__index.html]

Comprehensive identification of translation start sites by tetracycline-inhibited ribosome profiling — Supplementary Data 

# Comprehensive identification of translation start sites by tetracycline-inhibited ribosome profiling

## Supplementary Data

Supplementary Data

- Supplementary Data - Pdf file
- Supplementary Table 2-5 - xlsx file
