# Supplementary material for: Comprehensive identification of translation start sites by tetracycline-inhibited ribosome profiling
Source: DNA Res. 2016 Mar 23;23(3):193–201. doi: 10.1093/dnares/dsw008 (PMC4909307; doi:10.1093/dnares/dsw008)
Supplement: Supplementary Data [file supp_dsw008_dsw008supp.pdf]

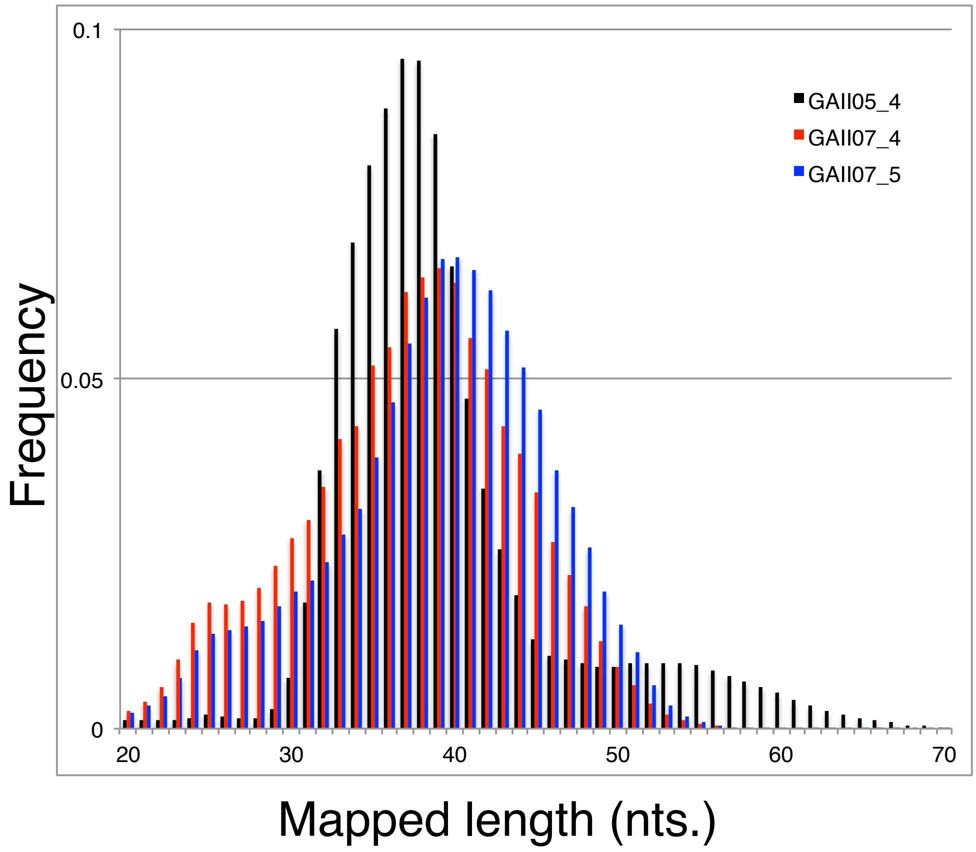

Figure S1. Length-distribution of TetRP reads mapped to CDS regions. Frequency of reads in each length is shown for three TetRP libraries. Total frequency for each library as set to 1.

```
           -35                   -10
base-lacZ  TACACT ttatgcttccggctcg TATAAT gtgtggaattgg
[GGATCCgtcgacctgcagccaagcttgagtaggacaaatccgccgagcttcgacgagattttcaggagctaaggaagctaaaATGgagaaaaaaatcact]
ggagatcccgtcgttttacaacgtcgtgactgggaaaaccctggcgttacccaacttaatcgccttgcagcacatccccctttcgccagctggcgtaatagcgaagaggcccgcacc
gatcgcccttcccaacagttgcgcagcctgaatggcgaatggcgctttgcctggtttccggcaccagaagcggtgccggaaagctggctggagtgcgatcttcctgaggccgatact
gtcgtcgtcccctcaaactggcagatgcacggttacgatgcgcccatctacaccaacgtaacctatcccattacggtcaatccgccgtttgttcccacggagaatccgacgggttgt
tactcgctcacatttaatgttgatgaaagctggctacaggaaggccagacgcgaattatttttgatggcgttaactcggcgtttcatctgtggtgcaacgggcgctgggtcggttac
ggccaggacagtcgtttgccgtctgaatttgacctgagcgcatttttacgcgccggagaaaaccgcctcgcggtgatggtgctgcgttggagtgacggcagttatctggaagatcag
gatatgtggcggatgagcggcattttccgtgacgtctcgttgctgcataaaccgactacacaaatcagcgatttccatgttgccactcgctttaatgatgatttcagccgcgctgta
ctggaggctgaagttcagatgtgcggcgagttgcgtgactacctacgggtaacagtttctttatggcagggtgaaacgcaggtcgccagcggcaccgcgcctttcggcggtgaaatt
atcgatgagcgtggtggttatgccgatcgcgtcacactacgtctgaacgtcgaaaacccgaaactgtggagcgccgaaatcccgaatctctatcgtgcggtggttgaactgcacacc
gccgacggcacgctgattgaagcagaagcctgcgatgtcggtttccgcgaggtgcggattgaaaatggtctgctgctgctgaacggcaagccgttgctgattcgaggcgttaaccgt
cacgagcatcatcctctgcatggtcaggtcatggatgagcagacgatggtgcaggatatcctgctgatgaagcagaacaactttaacgccgtgcgctgttcgcattatccgaaccat
ccgctgtggtacacgctgtgcgaccgctacggcctgtatgtggtggatgaagccaatattgaaacccacggcatggtgccaatgaatcgtctgaccgatgatccgcgctggctaccg
gcgatgagcgaacgcgtaacgcgaatggtgcagcgcgatcgtaatcacccgagtgtgatcatctggtcgctggggaatgaatcaggccacggcgctaatcacgacgcgctgtatcgc
tggatcaaatctgtcgatccttcccgcccggtgcagtatgaaggcggcggagccgacaccacggccaccgatattatttgcccgatgtacgcgcgcgtggatgaagaccagcccttc
ccggctgtgccgaaatggtccatcaaaaaatggctttcgctacctggagagacgcgcccgctgatcctttgcgaatacgcccacgcgatgggtaacagtcttggcggtttcgctaaa
tactggcaggcgtttcgtcagtatccccgtttacagggcggcttcgtctgggactgggtggatcagtcgctgattaaatatgatgaaaacggcaacccgtggtcggcttacggcggt
gattttggcgatacgccgaacgatcgccagttctgtatgaacggtctggtctttgccgaccgcacgccgcatccagcgctgacggaagcaaaacaccagcagcagtttttccagttc
cgtttatccgggcaaaccatcgaagtgaccagcgaatacctgttccgtcatagcgataacgagctcctgcactggatggtggcgctggatggtaagccgctggcaagcggtgaagtg
cctctggatgtcgctccacaaggtaaacagttgattgaactgcctgaactaccgcagccggagagcgccgggcaactctggctcacagtacgcgtagtgcaaccgaacgcgaccgca
tggtcagaagccgggcacatcagcgcctggcagcagtggcgtctggcggaaaacctcagtgtgacgctccccgccgcgtcccacgccatcccgcatctgaccaccagcgaaatggat
ttttgcatcgagctgggtaataagcgttggcaatttaaccgccagtcaggctttctttcacagatgtggattggcgataaaaaacaactgctgacgccgctgcgcgatcagttcacc
cgtgcaccgctggataacgacattggcgtaagtgaagcgacccgcattgaccctaacgcctgggtcgaacgctggaaggcggcgggccattaccaggccgaagcagcgttgttgcag
tgcacggcagatacacttgctgatgcggtgctgattacgaccgctcacgcgtggcagcatcaggggaaaaccttatttatcagccggaaaacctaccggattgatggtagtggtcaa
atggcgattaccgttgatgttgaagtggcgagcgatacaccgcatccggcgcggattggcctgaactgccagctggcgcaggtagcagagcgggtaaactggctcggattagggccg
caagaaaactatcccgaccgccttactgccgcctgttttgaccgctgggatctgccattgtcagacatgtataccccgtacgtcttcccgagcgaaaacggtctgcgctgcgggacg
cgcgaattgaattatggcccacaccagtggcgcggcgacttccagttcaacatcagccgctacagtcaacagcaactgatggaaaccagccatcgccatctgctgcacgcggaagaa
ggcacatggctgaatatcgacggtttccatatggggattggtggcgacgactcctggagcccgtcagtatcggcggaattccagctgagcgccggtcgctaccattaccagttggtc
tggtgtcaaaaataataataaccgggcaggggggatctccagcagccgcacgcggcgcatctcggctgttttggcggatgagagaagattttcagcctgatacagattaaatcagaa
cgcagaagcggtctgataaaacagaatttgcctggcggcagtagcgcggtggtcccacctgaccccatgccgaactcagaagtgaaacgccgtagcgccgatggtagtgtggggtct
ccccatgcgagagtagggaactgccaggcatcaaataaaacgaaaggctcagtcgaaagactgggcctttcgttttatctgttgtttgtcggtgaacgctctcctgagtaggacaaa
tccgccgggagcggatttgaacgttgcgaagcaacggcccggagggtggcgggcaggacgcccgccataaactgccaggcatcaaattaagcagaaggccatcctgacggatggcct
ttttgcgtttctacaaactcttcctgtcgtcatatctacaagccatccccccacagatacggtaaactagcctcgtttttgcatcaggaaagcagctgttttggcggatgagagaag
attttcagcctgatacagattaaatcagaacgcagaagcggtctgataaaacagaatttgcctggcggcagtagcgcggtggtcccacctgaccccatgccgaactcagaagtgaaa
cgccgtagcgccgatggtagtgtggggtctccccatgcgagagtagggaactgccaggcatcaaataaaacgaaaggctcagtcgaaagactgggcctttcgttttatctgttgttt
gtcggtgaacgctctcctgagtaggacaaatccgccgggagcggatttgaacgttgcgaagcaacggcccggagggtggcgggcaggacgcccgccataaactgccaggcatcaaat
taagcagaaggccatcctgacggatggcctttttgcgtttctacaaactcttcctgtcgtcatatctacaagccatccccccacagatacggtaaactagcctcgtttttgcatcag
gaaagcagtcgggcagcggccgc

ftsH-lacZ  GGATCCtaagaggttaatcccTTGagtgacATGgcgaaaaacctatcc
potA-lacZ  GGATCCatccgttgcgtttacATGggacagagtaaaaaaTTGaataaacaaccgtcc
rfaQ-lacZ  GGATCCacaaaagagtcgctaGTGgaaaagccatttcgaaaaatcctggtcataaagATGcgatatcatgggtcc
trkG-lacZ  GGATCCgcaaggtggttataaTTGaaagaatatttagatATGaatacatctcattcc
yigE-lacZ  GGATCCcatccaggacctataATGgcgcatcagctactcattggtaaaggaatgatcaccTTGaatctcaaacggtcc
ybjO-lacZ  GGATCCcgcggcattgttttcATGgaagacgaaacgTTGggatttttttaagtcc
yedI-lacZ  GGATCCtaaaaatgagagggaATGcttTTGgccgggagtagttcc
```

Figure S2. DNA sequence of translation initiation regions and modified *lacZ* gene for gene translation fusions. Sequence from the *lac*UV5 promoter to terminator region of base-*lacZ* plasmid is shown. Promoter -35 and -10 sequences and initiation codon are capitalized. Sequence replaced with test regions is bracketed. Sequences of test regions are shown below, where BamHI site and possible initiating codons are capitalized.

```
LOCUS       Venus_Cm_franment        1638 bp ds-DNA
FEATURES             Location/Qualifiers
     misc_recomb     complement(726..759)
                     /gene="FRT1"
                     /product="FRT1-FLP site specific recombination"
                     /SECDrawAs="Region"
                     /label=FRT1-FLP site specific recombination(1)
                     /ApEinfo_label=FRT1-FLP site specific recombination
     misc_recomb     complement(1585..1618)
                     /gene="FRT1"
                     /product="FRT1-FLP site specific recombination"
                     /SECDrawAs="Region"
                     /label=FRT1-FLP site specific recombination
     CDS             1..717
                     /gene="Venus-GFP"
                     /SECDrawAs="Gene"
     CDS             906..1544
                     /gene="cat"
                     /SECDrawAs="Gene"
ORIGIN
        1 gtgagcaagg gcgaggagct gttcaccggg gtggtgccca tcctggtcga gctggacggc
       61 gacgtaaacg gccacaagtt cagcgtgtcc ggcgagggcg agggcgatgc cacctacggc
      121 aagctgaccc tgaagctgat ctgcaccacc ggcaagctgc ccgtgccctg gcccaccctc
      181 gtgaccaccc tgggctacgg cctgcagtgc ttcgcccgct accccgacca catgaagcag
      241 cacgacttct tcaagtccgc catgcccgaa ggctacgtcc aggagcgcac catcttcttc
      301 aaggacgacg gcaactacaa gacccgcgcc gaggtgaagt tcgagggcga caccctggtg
      361 aaccgcatcg agctgaaggg catcgacttc aaggaggacg gcaacatcct ggggcacaag
      421 ctggagtaca actacaacag ccacaacgtc tatatcaccg ccgacaagca gaagaacggc
      481 atcaaggcca acttcaagat ccgccacaac atcgaggacg gcggcgtgca gctcgccgac
      541 cactaccagc agaacacccc catcggcgac ggccccgtgc tgctgcccga caaccactac
      601 ctgagctacc agtccgccct gagcaaagac cccaacgaga agcgcgatca catggtcctg
      661 ctggagttcg tgaccgccgc cgggatcact ctcggcatgg acgagcttta caagtaagga
      721 attccgaagt tcctatactt tgtacagaat aggaacttca taccgggaag ccctgggcca
      781 acttttggcg aaaatgagac gttgatcggc acgtaagagg ttccaacttt caccataatg
      841 aaataagatc actaccgggc gtatttttg agttgtcgag attttcagga gctaaggaag
      901 ctaaaatgga gaaaaaaatc actggatata ccaccgttga tatatcccaa tggcatcgta
      961 aagaacattt tgaggcattt cagtcagttg ctcaatgtac ctataaccag accgttcagc
     1021 tggatattac ggccttttta aagaccgtaa agaaaaataa gcacaagttt tatccggcct
     1081 ttattcacat tcttgcccgc ctgatgaatg ctcatccgga attacgtatg gcaatgaaag
     1141 acggtgagct ggtgatatgg gatagtgttc acccttgtta caccgttttc catgagcaaa
     1201 ctgaaacgtt ttcatcgctc tggagtgaat accacgacga tttccggcag tttctacaca
     1261 tatattcgca agatgtggcg tgttacggtg aaaacctggc ctatttccct aaagggttta
     1321 ttgagaatat gtttttcgtc tcagccaatc cctgggtgag tttcaccagt tttgatttaa
     1381 acgtggccaa tatggacaac ttcttcgccc ccgttttcac catgggcaaa tattatacgc
     1441 aaggcgacaa ggtgctgatg ccgctggcga ttcaggttca tcatgccgtt tgtgatggct
     1501 tccatgtcgg cagatgctta atgaatacaa cagtactgcg atgagtggca gggcggggcg
     1561 taaggcgcgc catttaaatg aagtgaagtt cctatacttt gtacagaata ggaacttcga
     1621 atcttcggta gtccagcg
//
```

```
IVP1_VF GCGTAATACAGGAGTAAGCGCAGATGTTTCATGATTTACCGGGAGTTAAAGTGAGCAAGGGCGAGGAGC
IVP2_VF GCAGATGTTTCATGATTTACCGGGAGTTAAATAGAGCATTGGCTATTCTTGTGAGCAAGGGCGAGGAGC
IVP3_VF GCATTCTAAACAAACAGCGTAACTCGTTATTGTTTGCGATCTACAATATCGTGAGCAAGGGCGAGGAGC
IVP4_VF TGCCGATGTCGGCACCAGATGCACACAGTGCCAGTGATCAATATTGTGCGGTGAGCAAGGGCGAGGAGC
IVP5_VF CAGCGACAGCAACGGCAATCTCAAACCCTGGGAAGAAGCGGTATCAGGTTGTGAGCAAGGGCGAGGAGC
IVP6_VF TTTCTCTGTCGATGCTCTTCTGGGCCGAACTCCTCTGGATCATTACTCACGTGAGCAAGGGCGAGGAGC
IVP7_VF CGATTAAATTGTCGCATCGTGAGCGTAGATCTGCGAGGATACGCGCCTGCGTGAGCAAGGGCGAGGAGC
IVP1_VR AGGCTGTGAATACTCATGTATTCAGCCACCCTTAAAGAATAGCCAATGCTCGCTGGACTACCGAAGATTC
IVP2_VR TCGTCCTCACTTCAGGTAAGGCTGTGAATACTCATGTATTCAGCCACCCCGCTGGACTACCGAAGATTC
IVP3_VR TAAAGACGGAAGAGTAAACTTATTGGGCACCGCAATGGTGCCCAGTACATCGCTGGACTACCGAAGATTC
IVP4_VR CCTGATACCGCTTCTTCCCAGGGTTTGAGATTGCCGTTGCTGTCGCTGCACGCTGGACTACCGAAGATTC
IVP5_VR CTTATGTGCTTCCTCCGGGCCGACTTTTTGCCAGGAATCAAGGAAGCTGGCGCTGGACTACCGAAGATTC
IVP6_VR GAAACCTTGTGGCAAAGCAAATGACAACCCCGCCGCAGCGGGGTCAAGGACGCTGGACTACCGAAGATTC
IVP7_VR CAGGGTAGAAAAAGCGGTCACAATCTATTCTCGTGGTCATCGACGCAAAGCGCTGGACTACCGAAGATTC
```

Figure S3. Template and primer sequences for Venus gene fusion construction. Sequence of the Venus-cat fragment used as PCR template and primers used are shown. Cf. IVP1_VF and IVP1_VR were used to amplify fragment to create IVP1-Venus gene fusion in the chromosome.

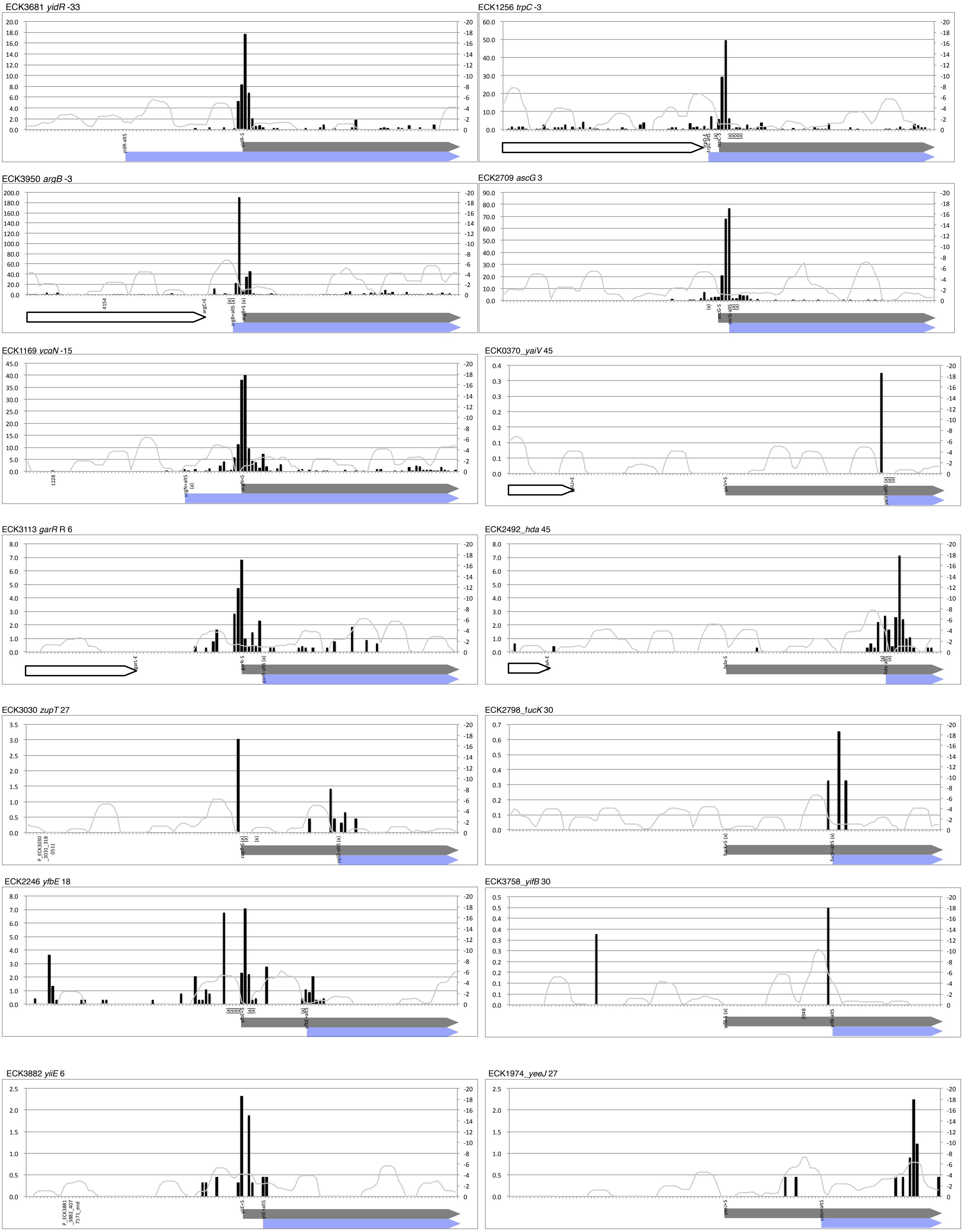

Figure S4(continues) TetRP signal around initiation region of genes which annotation had changed from 2006 to 2014, but not found in our analysis.

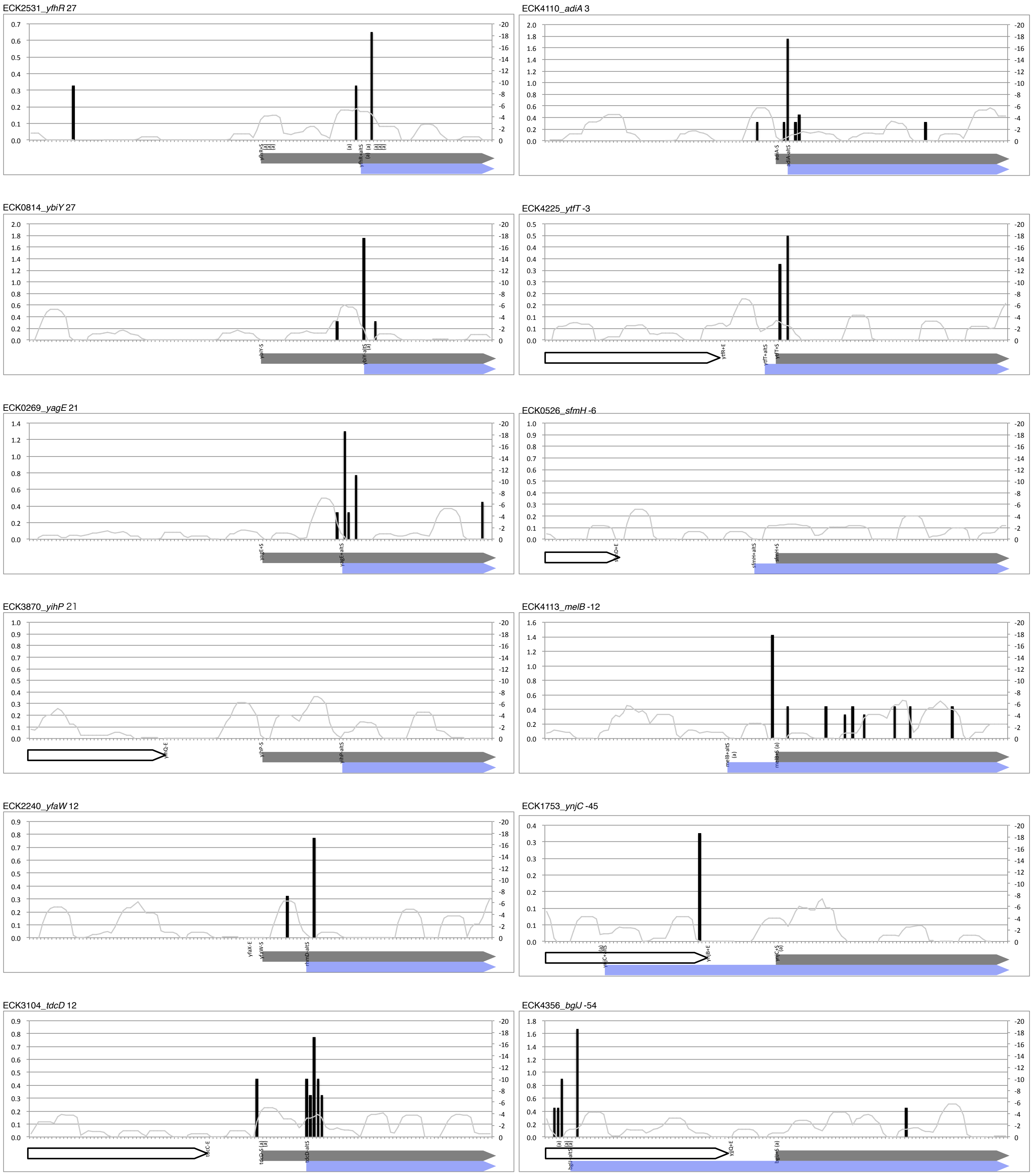

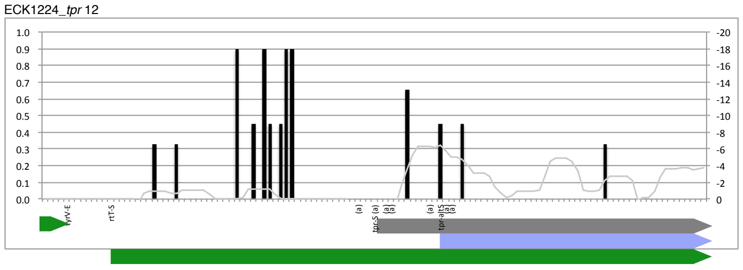

Figure S4 (continued) TetRP signal near initiation regions of genes with annotation changes from 2006 to 2014, but not re-annotated by TetRP analysis. Black bar indicating TetRP signal and gray line indicating hybridization stability with anti-ribosome binding site are same with Figure 3. Coding region starting from the 2006 (gray) and 2014 (blue) annotations are indicated as thick arrows and an upstream CDS or a stable RNA gene is sown by an thick white or thick green arrow, respectively. The gene name with S, altS, or E is shown in X-axis at the start of 2006 annotation, start of 2014 annotation, or end position of the gene, respectively, and an (a) is shown at a position where sequence reads giving the signal at the position ends with adenine.

ATG initiation

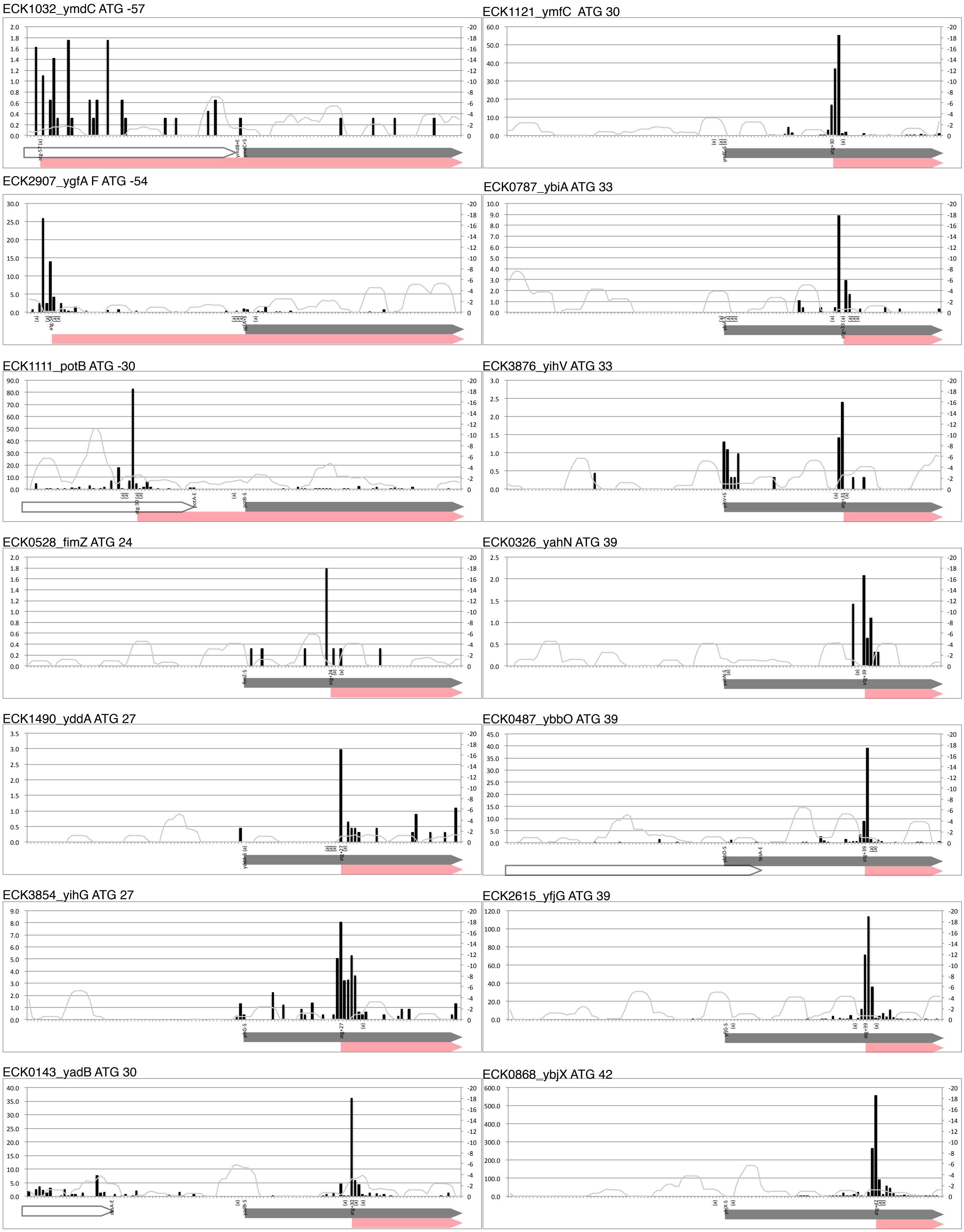

Figure S5 TetRP signal of gene-5'-part (continues)

ECK0293_ykgK ATG 48

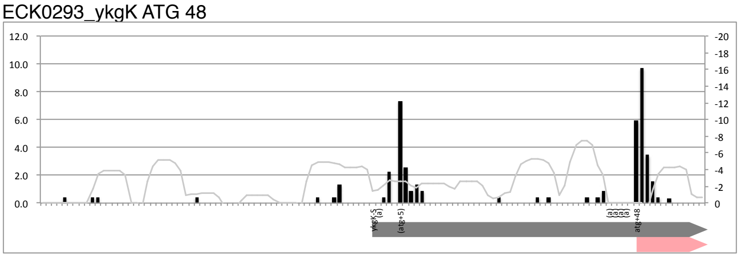

ECK3881_yiiD ATG 48

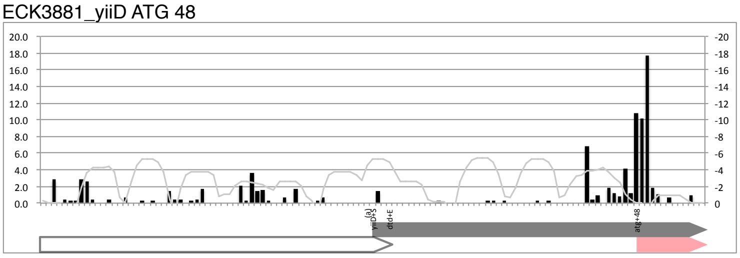

ECK3590_mtlR ATG 51

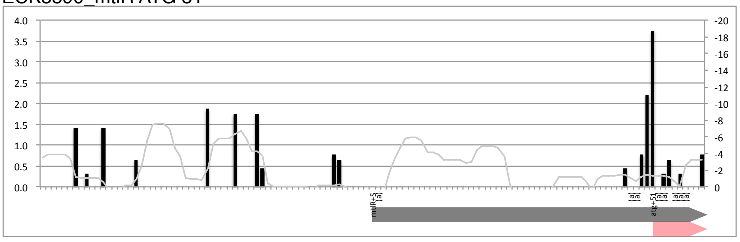

ECK4036_dinF ATG 54

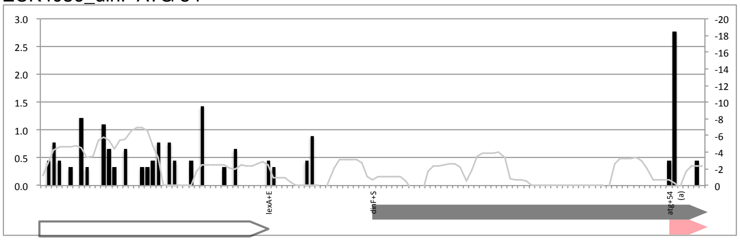

ECK3002_yqhC ATG 57

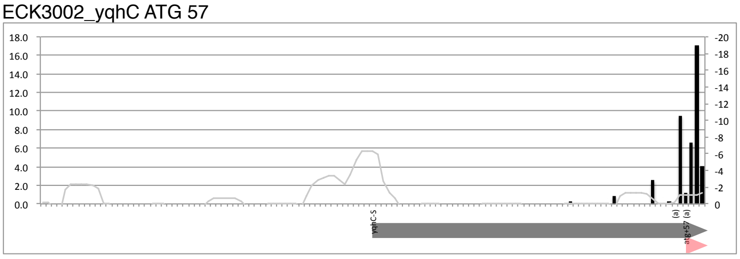

ECK2095_yegX ATG -9

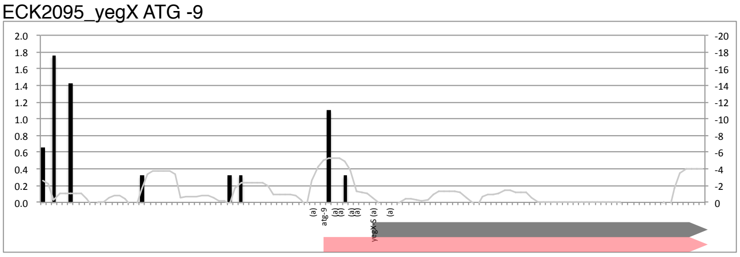

ECK3771_gpp ATG -9

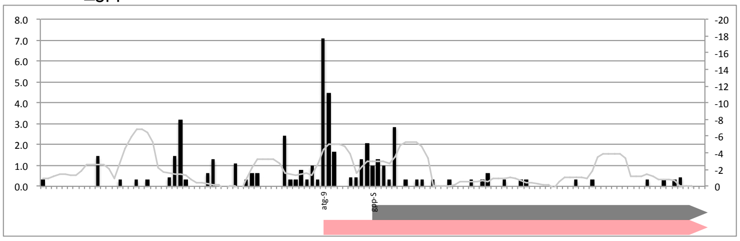

ECK1488_pqqL ATG 12

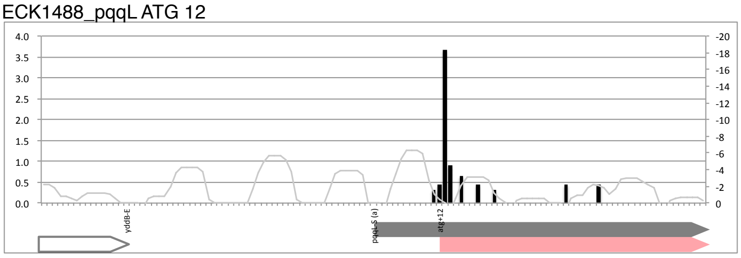

ECK1517_yneH ATG 12

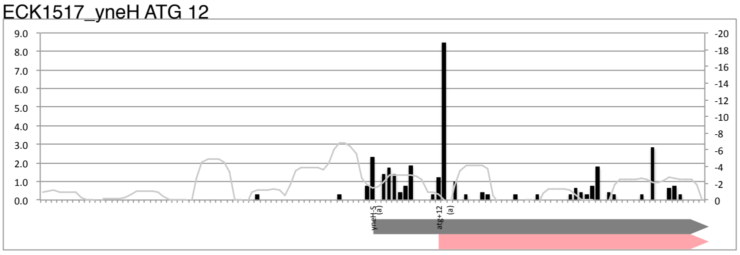

ECK1552_cspF ATG 12

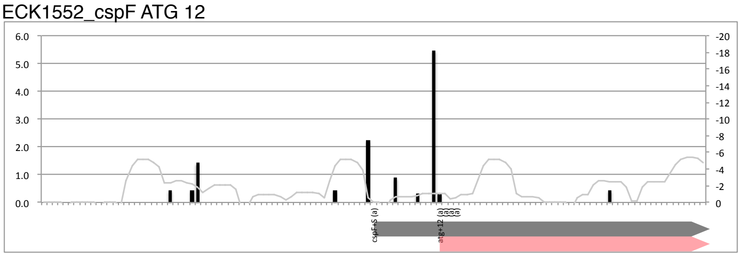

ECK4230_pmbA ATG 12

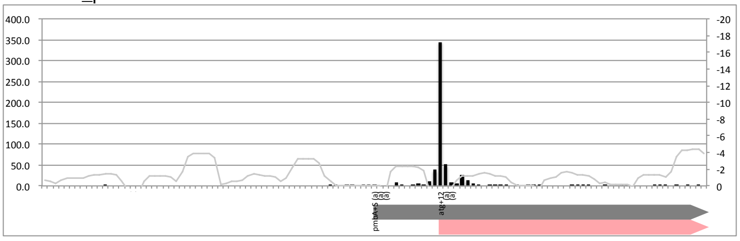

ECK3563_yiaJ ATG 15

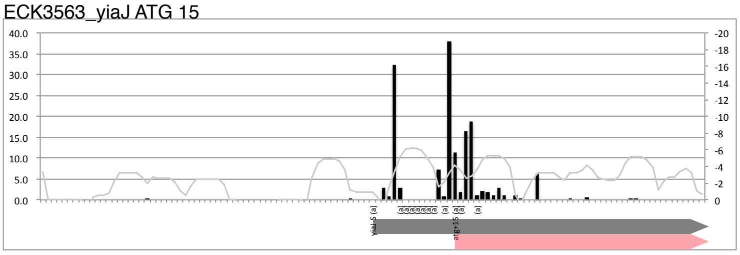

ECK0359_yaiF ATG 18

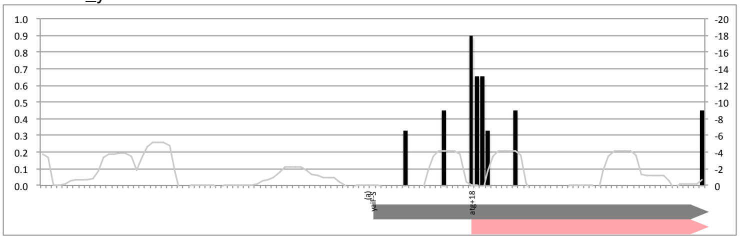

ECK1444_yncC ATG 18

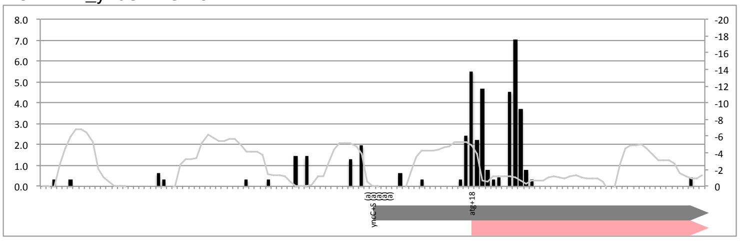

Figure S5 TetRP signal of gene-5'-part (continues)

ECK4041_yjbN ATG 18

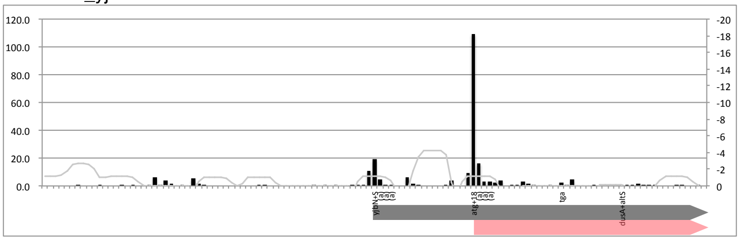

ECK2282_nuoA ATG 6

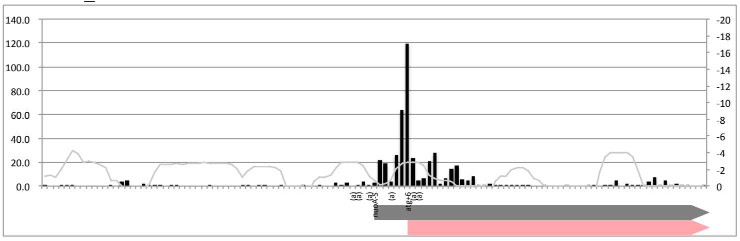

ECK0021_nhaR ATG 6

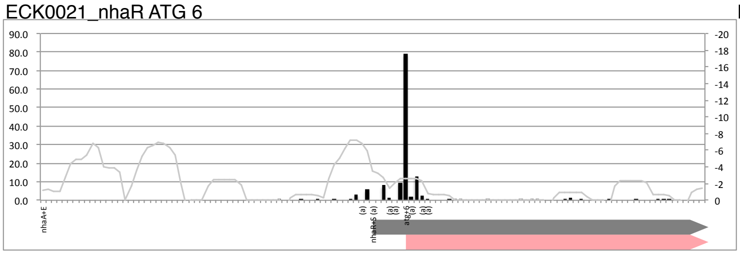

ECK0545_essD ATG 9

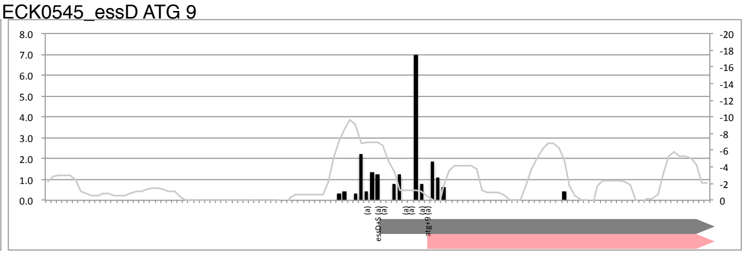

ECK0833_ybjH ATG 6

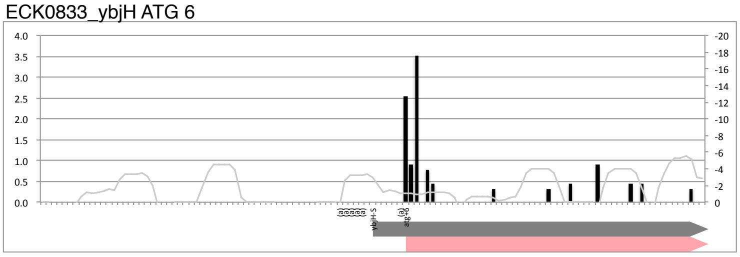

ECK1124_ymfE ATG 9

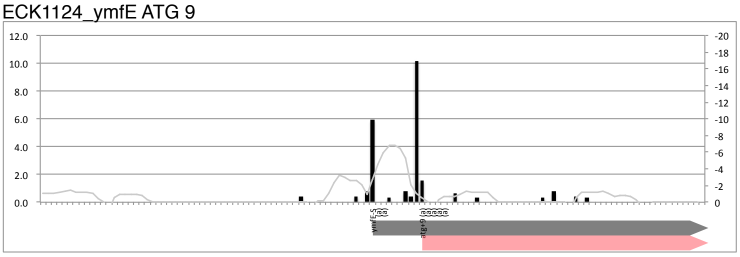

ECK1635_ydhA ATG 6

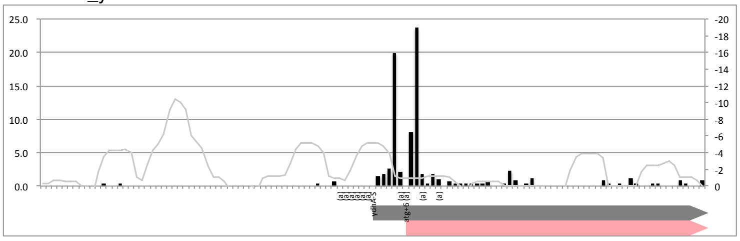

ECK1888_cheW ATG 9

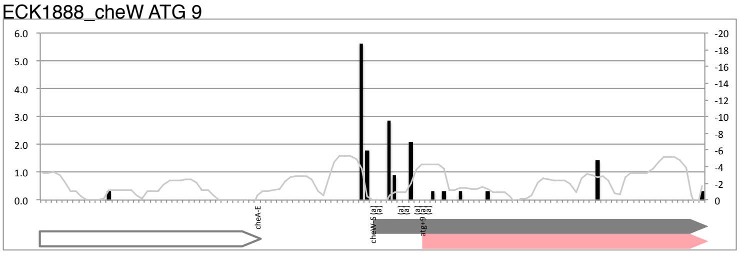

ECK2083_gatR ATG 6

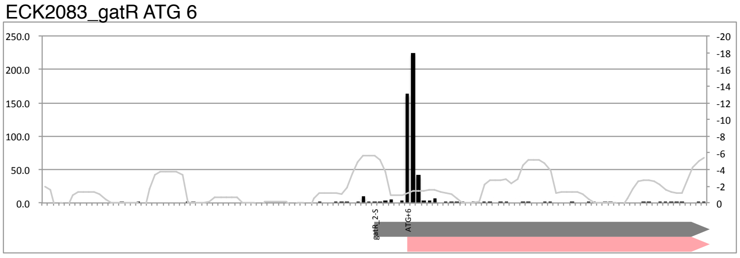

ECK3215_nanR ATG 9

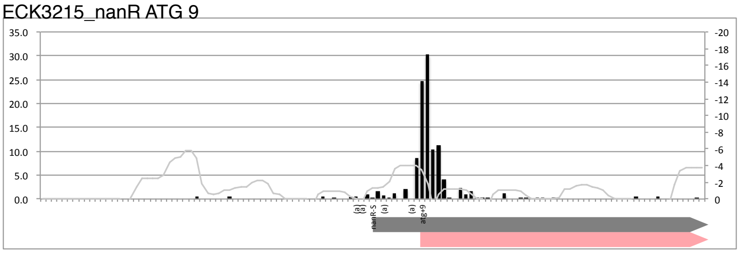

ECK2193_ccmA ATG 6

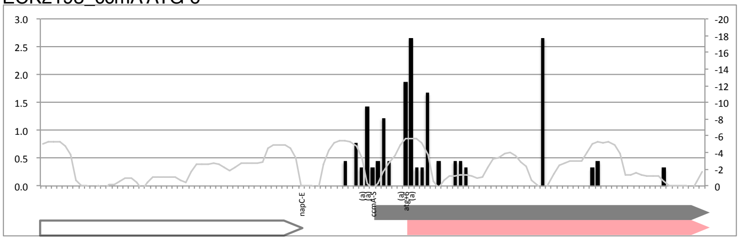

ECK3835_ubiD ATG 9

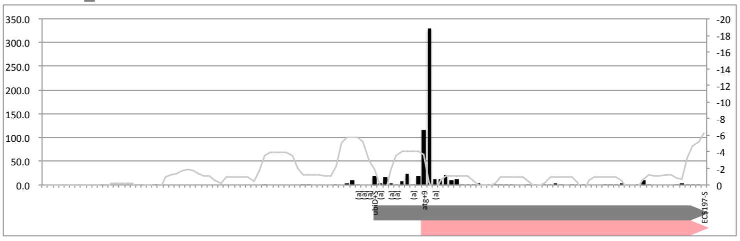

Figure S5 TetRP signal of gene-5'-part (continues)

GTG initiation

ECK2986_hybE GTG -39

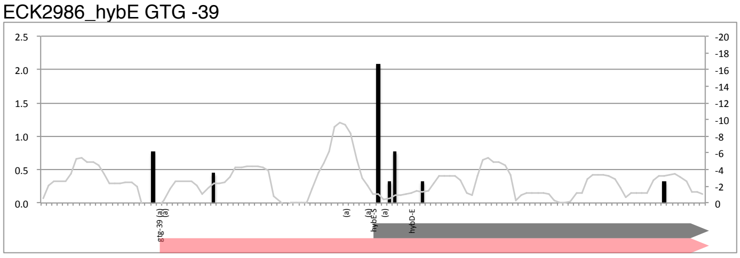

ECK3521_bcsE GTG 12

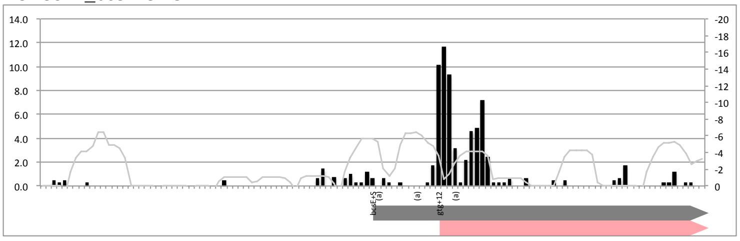

ECK3622_rfaQ GTG -36

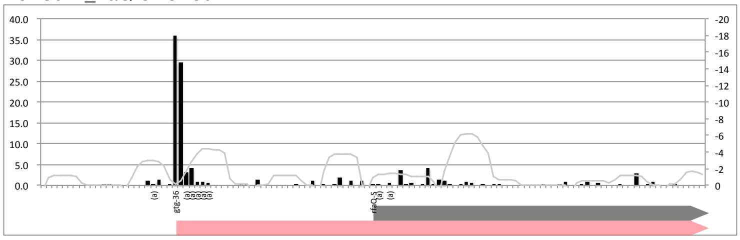

ECK1025_csgE GTG 24

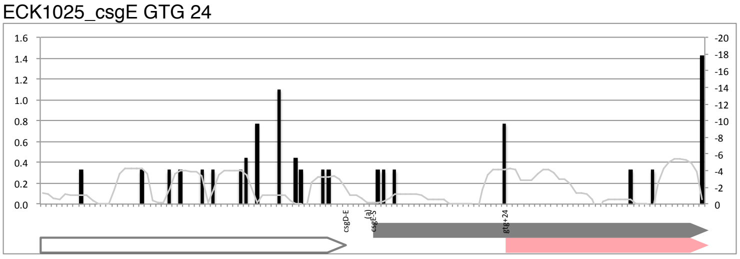

ECK2028_wbbI GTG -6

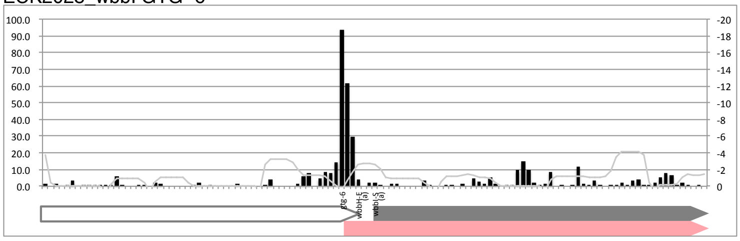

ECK0932_ycbT GTG 27

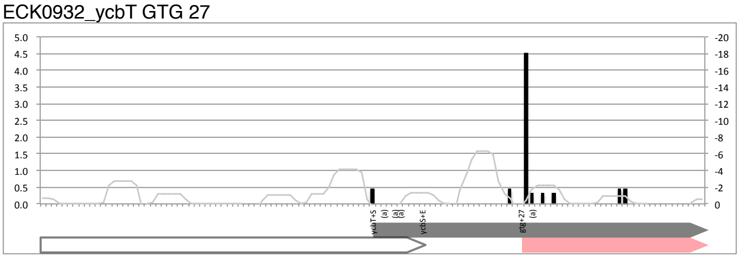

ECK3664_emrD GTG -6

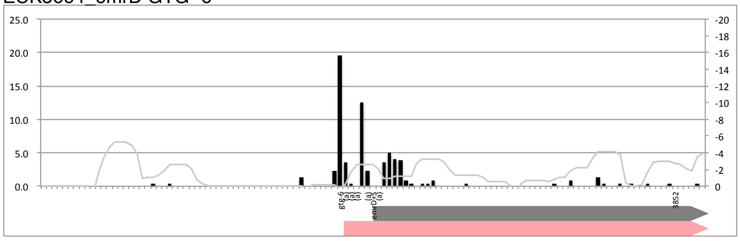

ECK3129_agaI GTG 54

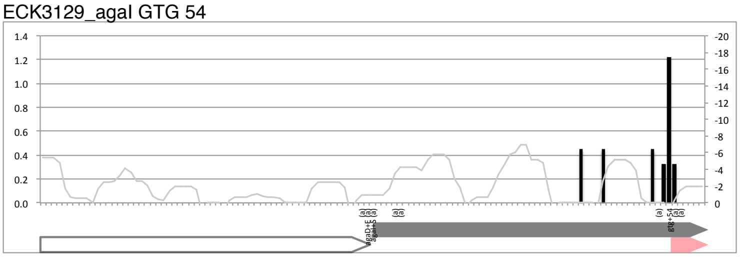

ECK2896_bglA GTG 6

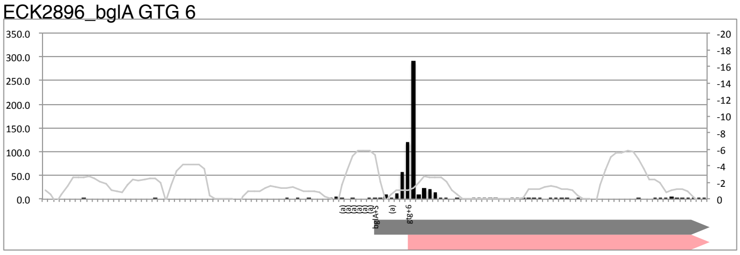

ECK0615_crcA GTG 6

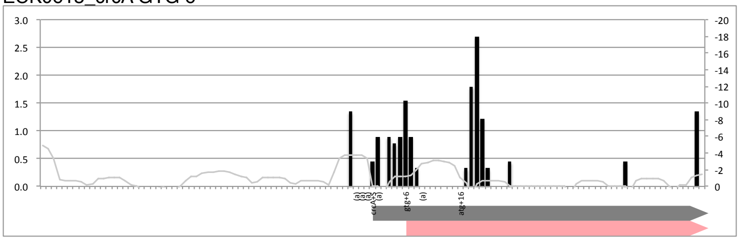

Figure S5 TetRP signal of gene-5'-part (continues)

TTG initiation

ECK1362_trkG TTG -18

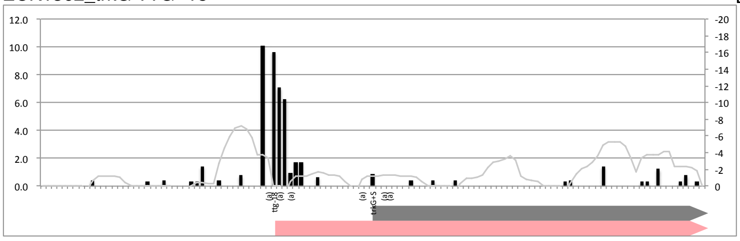

ECK0849_ybjO TTG 15

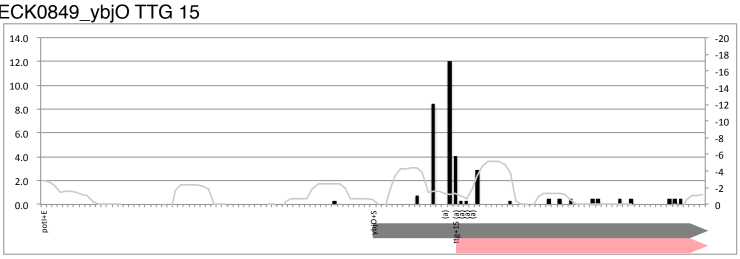

ECK4105_basS TTG -9

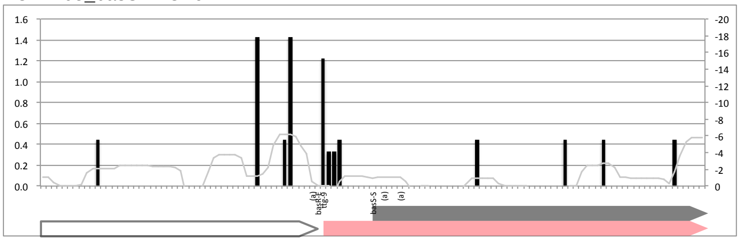

ECK1112_potA TTG 18

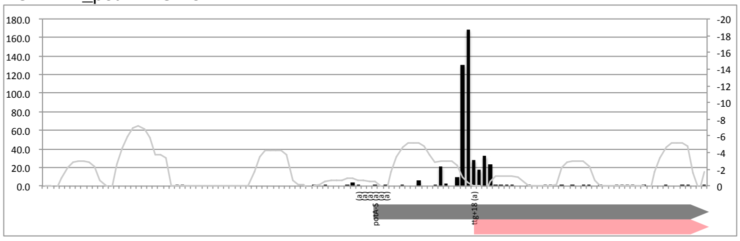

ECK3167_ftsH TTG -9

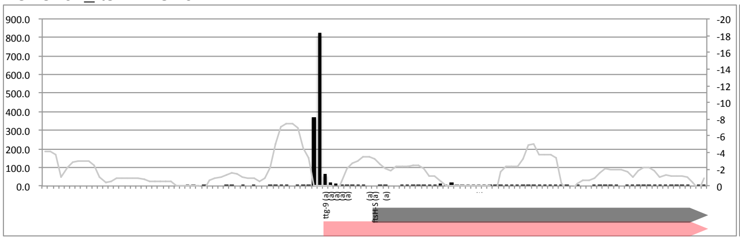

ECK3809_yigE TTG 39

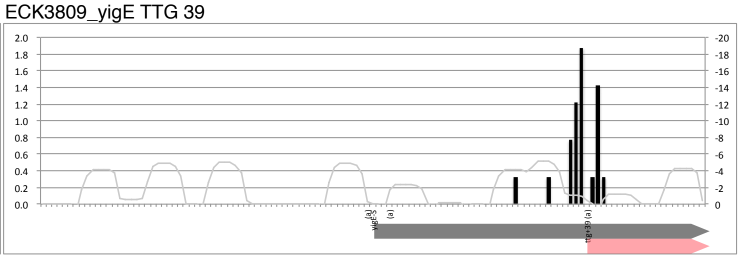

ECK1956_yedI TTG 6

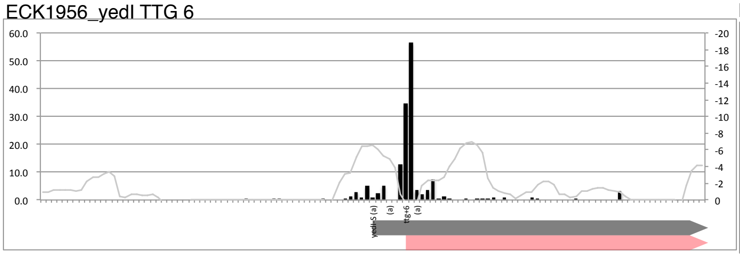

ECK1123_ymfD TTG 12

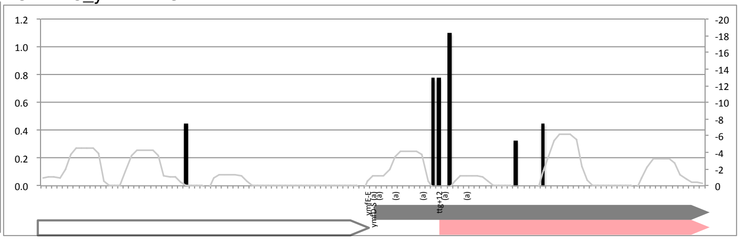

ECK2739_surE TTG 9

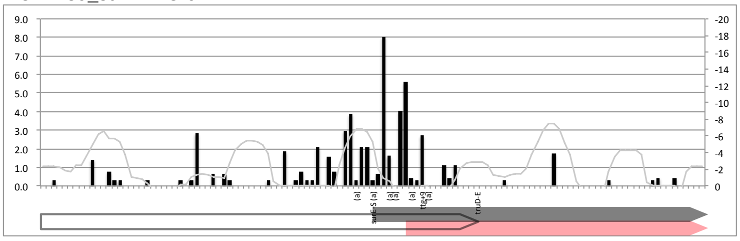

Figure S5 TetRP signal of gene-5'-part (continues)

CTG initiation

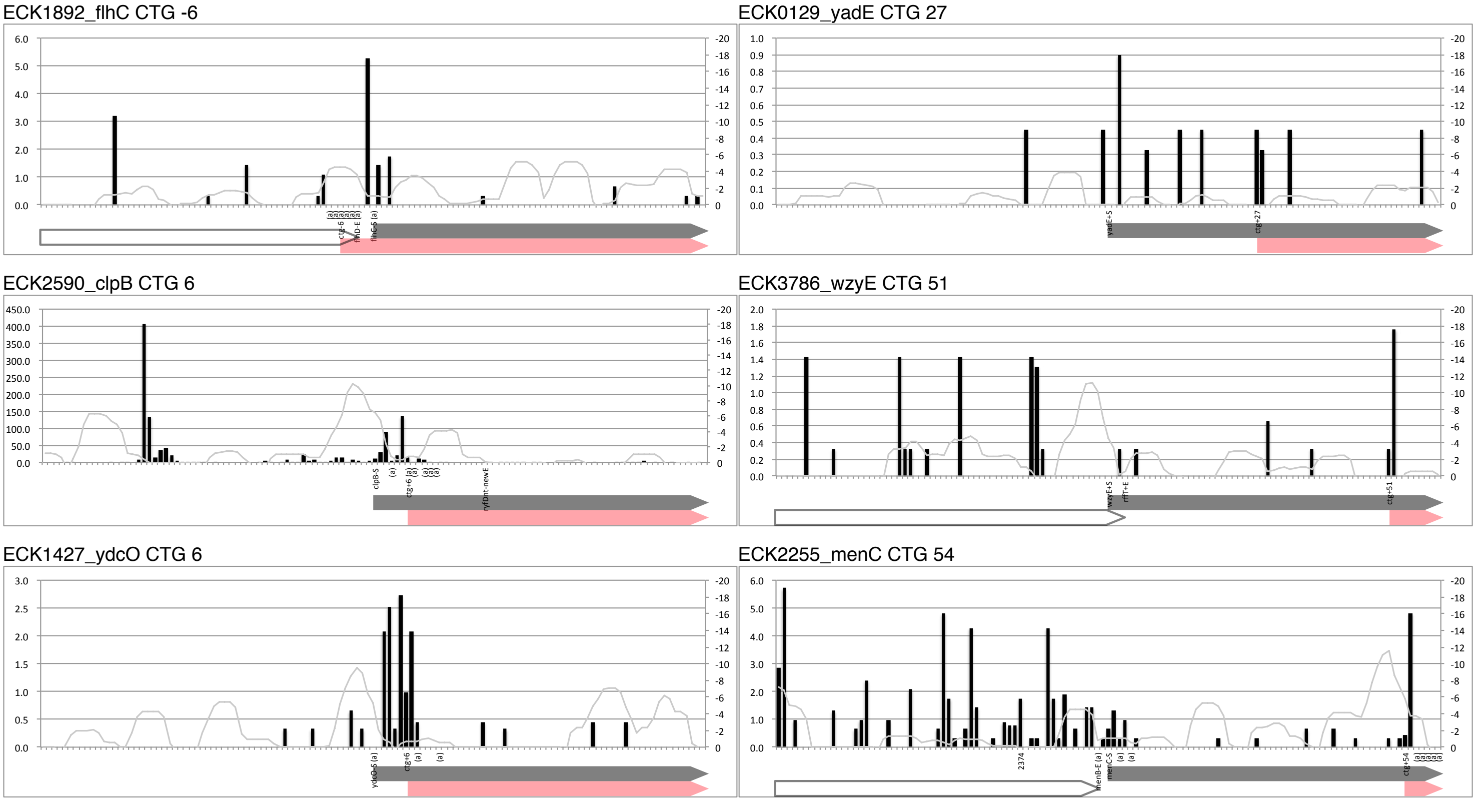

Figure S5 TetRP signal of gene-5'-part (continued)

TetRP signals near initiation regions of genes found to have new start sites by TetRP but unchanged from 2006 to 2014 annotation. Notations are essentially the same with Figure S4. Coding region by TetRP is shown as thick red arrow and its start position is indicated by the starting codon and distance (nucleotides) from the start position in 2006 annotation.

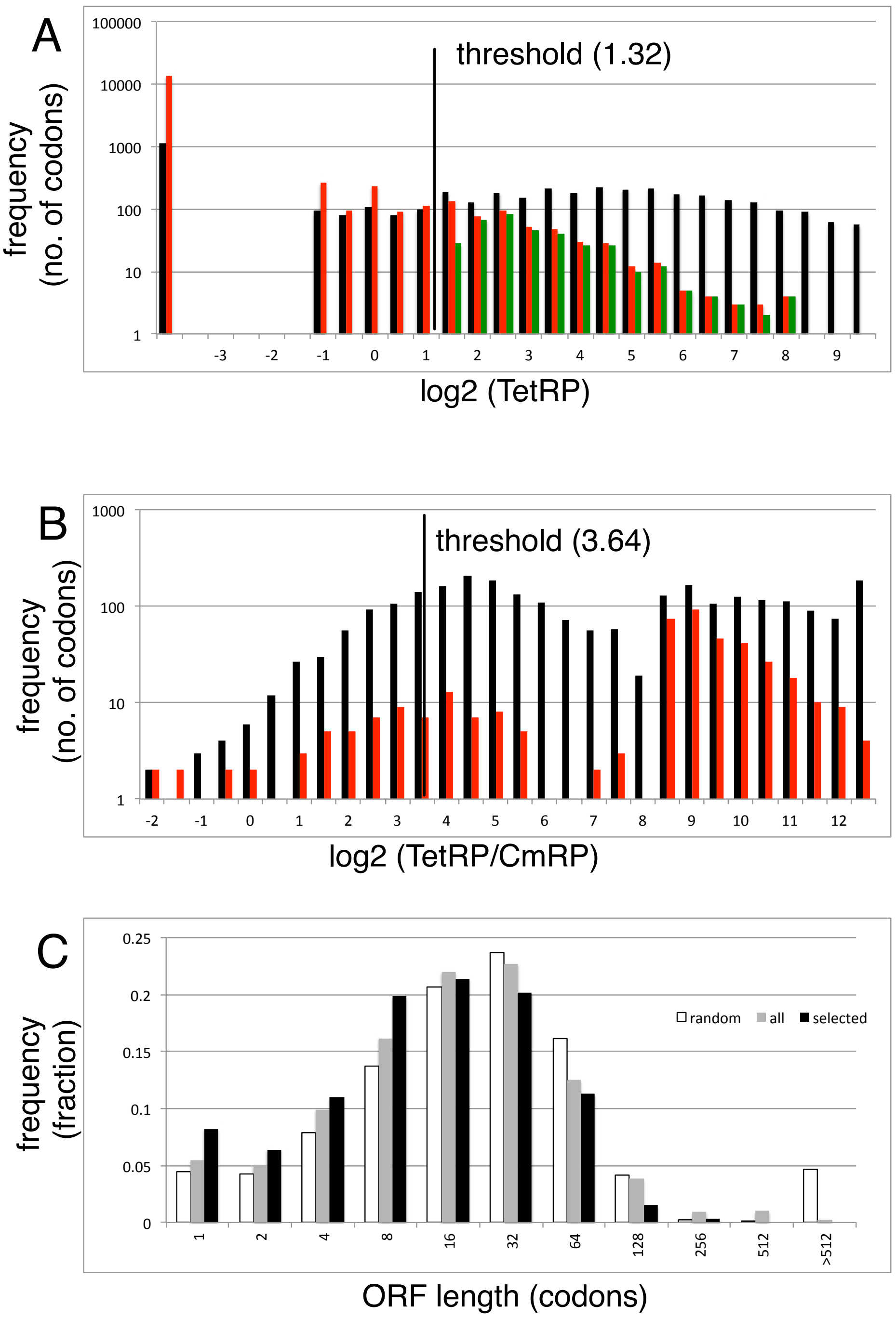

Figure S6 Selection of possible initiation sites and length distribution of small ORFs with the start sites within intergenic regions. A) Distribution of TetRP value of all initMets (black), all betMets (red), and 360 selected betMets (green) are indicated. X-axis value indicated -3: -3≥ x, -2: -3 < x ≤ –2, -1: -2 < x ≤ –1, etc. Threshold of the first selection is marked with vertical bar. B) Distribution of TetRP/CmRP values of initMets (black) and betMets (red) which passed the first selection. Threshold of the second selection is marked with vertical bar. C) Length distribution of predicted translation products from intergenic start sites. Length distribution of ORFs by translation of randomized genetic code (white), of predicted products from all betMets (gray) and from selected 360 initiation sites (black) are shown.

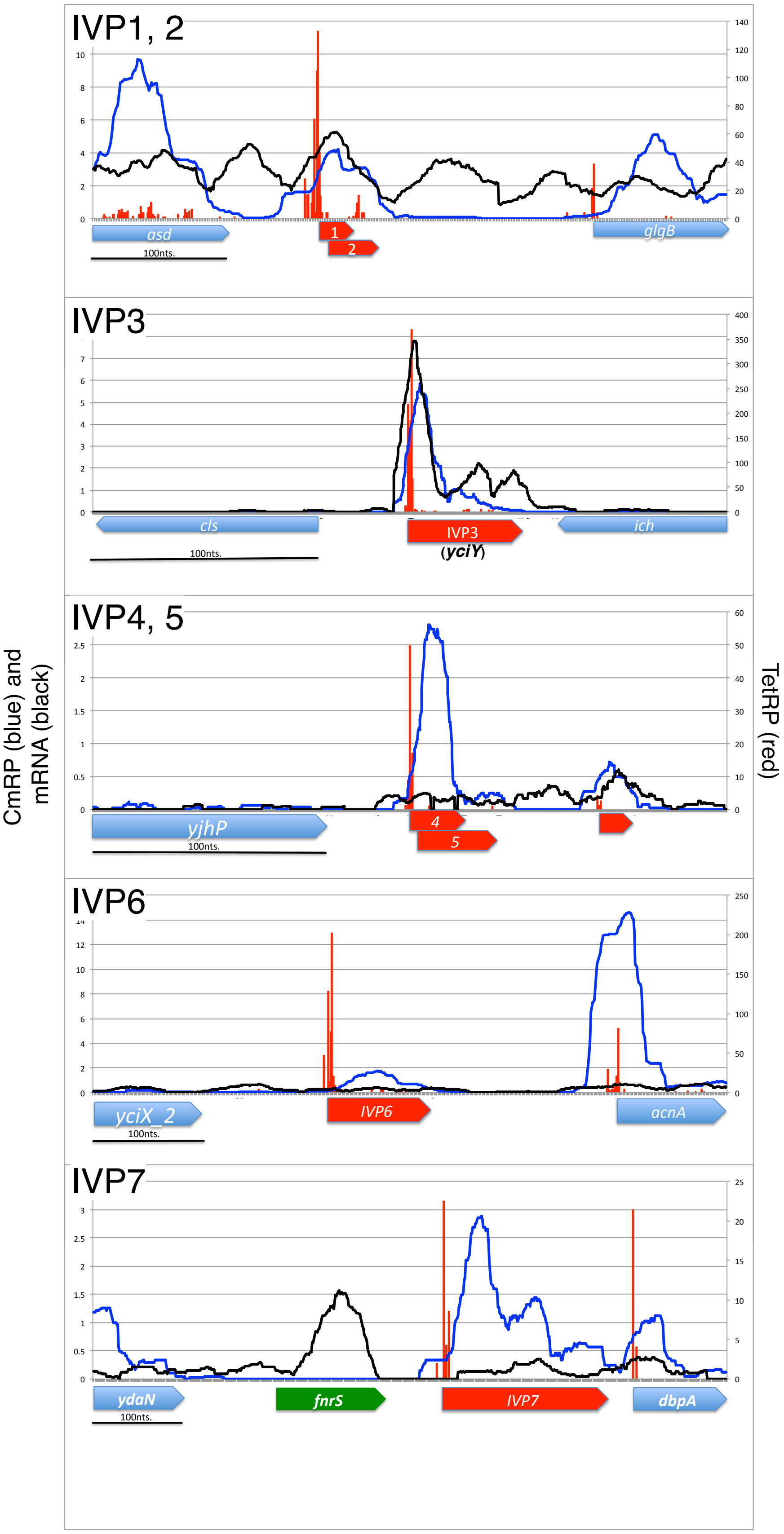

Figure S7 Transcription and RP patterns of sORFs examined with Venus gene fusions. Annotations are essentially the same as upper panel of Figure 5. sORF region, adjacent coding genes and non-coding gene are in thick red, blue and green arrows, respectively.

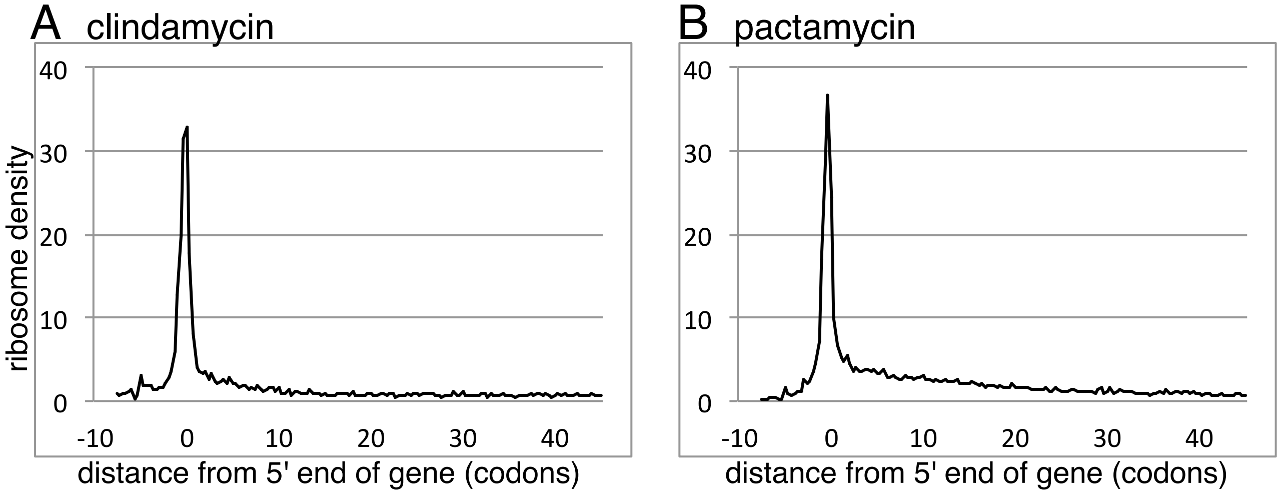

Figure S8. Patterns near 5' end of coding region of genes in clindamycin and pactamycin inhibited RP. Same as Figure 1 b and c, except that RP samples were taken after clindamycin (A) or pactamycin (B) treatment.

Table S1 Sequenced samples and number of reads mapped to CDS

Name	source strain	library*1	CDS reads*2
GAII05_3	BW25113 *smpB*	Cm-RP	2,538,972
GAII05_5	BW25113	Cm-RP	3,959,115
GAII_9_4	BW25113	Cm-RP	2,124,985
GAII06_1	BW25113 *smpB*	RNA-seq	5,127,408
GAII05_8	BW25113	RNA-seq	1,611,231
GAII_8_10	BW25113	RNA-seq	5,967,668
GAII05_4	BW25113	TetRP	1,027,370
GAII07_4	BW25113	TetRP	3,357,792
GAII07_5	BW25113	TetRP	4,498,133

*1 Type of library used for sequencing

*2 Total number of the sequence reads mapped to CDSs
